# Binding of undamaged double stranded DNA to vaccinia virus uracil-DNA Glycosylase

**DOI:** 10.1186/s12900-015-0037-1

**Published:** 2015-06-02

**Authors:** Norbert Schormann, Surajit Banerjee, Robert Ricciardi, Debasish Chattopadhyay

**Affiliations:** Department of Medicine, University of Alabama at Birmingham, Birmingham, AL 35294 USA; Northeastern Collaborative Access Team and Department of Chemistry and Chemical Biology, Cornell University, Argonne, Chicago, IL 60439 USA; Department of Microbiology, School of Dental Medicine, Abramson Cancer Center, University of Pennsylvania, Philadelphia, PA 19104 USA

**Keywords:** Protein-DNA structure, Non-specific DNA, Early DNA recognition complex, Uracil-DNA glycosylase, Poxvirus

## Abstract

**Background:**

Uracil-DNA glycosylases are evolutionarily conserved DNA repair enzymes. However, vaccinia virus uracil-DNA glycosylase (known as D4), also serves as an intrinsic and essential component of the processive DNA polymerase complex during DNA replication. In this complex D4 binds to a unique poxvirus specific protein A20 which tethers it to the DNA polymerase. At the replication fork the DNA scanning and repair function of D4 is coupled with DNA replication. So far, DNA-binding to D4 has not been structurally characterized.

**Results:**

This manuscript describes the first structure of a DNA-complex of a uracil-DNA glycosylase from the poxvirus family. This also represents the first structure of a uracil DNA glycosylase in complex with an undamaged DNA. In the asymmetric unit two D4 subunits bind simultaneously to complementary strands of the DNA double helix. Each D4 subunit interacts mainly with the central region of one strand. DNA binds to the opposite side of the A20-binding surface on D4. Comparison of the present structure with the structure of uracil-containing DNA-bound human uracil-DNA glycosylase suggests that for DNA binding and uracil removal D4 employs a unique set of residues and motifs that are highly conserved within the poxvirus family but different in other organisms.

**Conclusion:**

The first structure of D4 bound to a truly non-specific undamaged double-stranded DNA suggests that initial binding of DNA may involve multiple non-specific interactions between the protein and the phosphate backbone.

**Electronic supplementary material:**

The online version of this article (doi:10.1186/s12900-015-0037-1) contains supplementary material, which is available to authorized users.

## Background

Repair of damages in DNA is an essential cellular process regulated by specialized molecular machinery. Base excision repair (BER) pathway [1] for repair of small lesions is initiated by monofunctional DNA glycosylases. These enzymes use a water molecule as a nucleophile to cleave the N-glycosidic bond between the target base and deoxyribose, releasing the damaged base and leaving an apurinic/apyrimidinic (AP) site [2]. The BER pathway for the removal of uracil (Ura), which arises in DNA from deamination of cytosine (Cyt) or incorporation of dUTP during DNA synthesis, is initiated by uracil-DNA glycosylases (UDGs) [3]. UDGs are divided in different families based on their substrate specificity [4]. Members of family I-V share similar structure and motifs. Family I UDGs, which are also known as UNGs, specifically excise uracil from single-stranded DNA (*ss*DNA) and double-stranded DNA (*ds*DNA) with the preference *ss*U > *ds*U:G > *ds*U:A [5]. UNGs are ubiquitous enzymes that use highly conserved motifs for DNA binding, uracil recognition and excision.

The reaction mechanism for UNGs has been elucidated from a series of elegant structural studies in which wild-type and catalytically inactive mutant UNGs were captured in the DNA-bound form at various stages of action [6–12]. For detection of uracil in DNA, UNGs use a ‘pinch-push-pull’ mechanism, which involves a multi-step base flipping process. In the first step, serine and proline residues in three different loops (the ‘Pro-rich loop’, the ‘Gly-Ser loop’ and the ‘Leu-intercalation loop’) lead to a slight bending of the DNA through compression (‘pinch’) of the backbone. In the second step, a conserved leucine residue of the ‘Leu-intercalation loop’ penetrates into the minor groove and the DNA becomes fully bent and kinked leading to flipping of the uracil base (‘push’). The uracil nucleotide interacts with the ‘uracil recognition pocket’ where cleavage of the glycosidic bond takes place, and in the final step, the leucine residue is retracted (‘pull’). Two catalytic residues, an aspartic acid and a histidine, are found invariant in all UNGs (Table 1).

**Table 1:** Comparison of motifs for DNA binding and catalysis

	1FLZ (EcUNG)	1SSP (hUNG)	4QCB (vUNG)
Catalytic water-activating loop	62-GQDPYH-67	143-GQDPYH-148	66-GIDPYP-71
Pro-rich loop	84-AIPPS-88	165-PPPPS-169	84-FTKKS-88
Uracil specificity β-strand	120-LLLN-123	201-LLLN-204	117-IPWN-120
Gly-Ser loop	165-GS-166	246-GS-247	160-KT-161
Leu intercalation loop	187-HPSPLSVYR-195	268-HPSPLSAHR-276	181-HPAARDR-187
Active site residues	D64, Y66, F77, N123, H187, L191	D145, Y147, F158, N204, H268, L272	D68, Y70, F79, N120, H181, R185

Motifs as previously listed [13] are updated based on structural superimposition using the new D4-DNA complex structure

UNGs of poxviruses are, however, exceptional and most diverse members of this family. The motifs used by these enzymes are fully conserved in all orthopoxviruses but differ significantly from their counterparts in other organisms (Additional file 1: Table S1) [4, 13–15]. More importantly, UNGs of poxviruses assume a novel and essential role in viral replication and serve as an intrinsic component of the replication machinery. In the prototypic poxvirus Vaccinia, UNG (known as D4) binds to another viral protein A20 to form the heterodimeric processivity factor. A20 also binds to the DNA polymerase E9 and thereby tethers D4 to E9 [15–20] and assembles the heterotrimeric (D4:A20:E9) core of the processive polymerase complex. In the absence of D4, Vaccinia E9 synthesizes only short (<10 nucleotides) stretches of DNA [18, 21]. This essential role of D4 in supporting processive DNA synthesis is not dependent on its catalytic (glycosylase) activity [15].

**Fig. 1:**
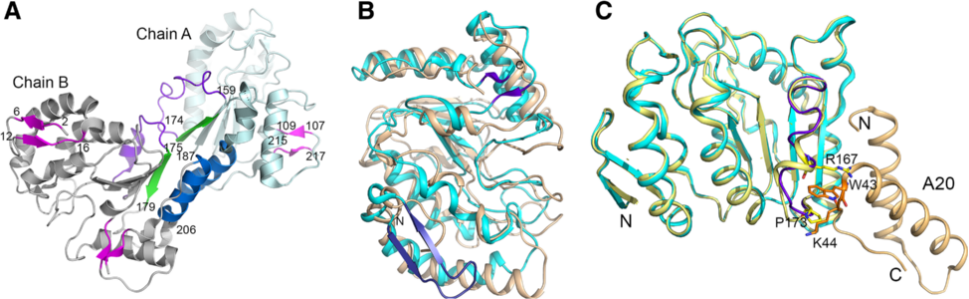
Structure of D4. **a.** Overall structure of D4. Cartoon diagram shows the three dimensional structure of D4. The A and B subunits from the free-D4 structure (*4dof*) are displayed and labeled. C-terminal strand of the central β-sheet is colored green. Additional β sheets seen in D4 structures are colored magenta; amino acid residues forming the strands are labeled. Structural elements that are involved in homodimer interactions in different D4 structures are painted in blue, violet and green. **b.** Comparison with human UNG (hUNG). Cartoon drawing shows superimposition of the structure of substrate-free hUNG (*1akz*) in wheat color and D4 (*4dof* subunit **a)** in cyan. Overall structural fold is very similar for both proteins. The N and C-terminal additional β sheets in D4 are colored dark. N and C-terminal ends of D4 are labeled. **c.** A20 binding on D4. Cartoon drawing shows the A20 binding site on D4 structure. Structure of D4 (*4dof*, subunit A, cyan) was superimposed on the D4 chain A of D4:A20 complex (*4od8,* yellow). A20 segment from 4*od8* is shown in wheat color. Amino acid residues Arg167 and Pro173 of D4 and Trp43 and Lys44 of A20 play important role in the binding. These residues are shown as stick models. N and C-terminus of A20 are labeled

Crystal structures of D4 have been reported in the free form [13,22; *2owr*^1^*,*^1^*2owq, 3 nt7, 4dof, 4dog*] and as a non-productive^2^^2^ uracil complex [14; *4lzb*]. Three dimensional structure of D4 retains the overall fold of UNG proteins- a central 4-stranded parallel β-sheet with α-helices on both sides (Fig. 1). There are two additional β-sheets in D4 (residues 2-6/12-16 and 107-109/215-217) not seen in any other UNG (Figs. 1a, b). Although the UNG-specific motifs are very different, the architecture of the uracil-binding pocket in D4 is remarkably similar to other UNGs and five of the six residues forming the pocket are identical. These residues include the catalytic Asp68 and His181, which are appropriately positioned for binding uracil and cleavage of the glycosidic bond [14]. Considering the unique properties of poxvirus UNGs, it should be highly interesting to understand how these proteins adapt to utilizing the altered motifs for binding DNA, and recognizing and excising uracil.

All crystal structures of free-D4 and the structure of the uracil complex feature a characteristic dimeric packing. Although specific protein-protein interactions between D4 subunits vary to some extent the dimer interface is very similar in these structures [13, 14, 22]. This homodimer interface is formed by residues located in the C-terminal β-strand of the central β-sheet in each subunit, a flexible loop/short helix (residues 161–173) and a long helix (187–206) located near the C-terminus. The first site, which is located in a loop connecting the two C-terminal β-strands, represents one of the most flexible areas in the D4 structures (Fig. 1a). At high protein concentration D4 utilizes the same interface for dimerization in solution. However, when A20 is present, very specific interaction at this surface leads to the formation of a D4:A20 heterodimer (Fig. 1c) [19, 20]. Cumulative structural and biochemical evidences indicate that in the processive DNA polymerase complex, D4 remains catalytically active and possibly continuously scans and removes uracil from newly synthesized DNA [18–20]. Details of D4’s ability to bind and scan DNA, its enzymatic function and substrate specificity have been reported [22, 23]. However, structural characterization of the DNA-binding site(s) on D4 is missing.

Here, we report the first detailed view of D4-DNA interactions from a crystal structure of D4 in complex with a non-specific undamaged *ds*DNA. The structure of this complex for the first time reveals the DNA-binding interactions by an UNG from the poxvirus family. It also represents the first three-dimensional structure of a UNG bound to a truly undamaged *ds*DNA and provides a representative snapshot of the initial interaction between UNG and DNA. We also present a structural comparison of D4 and human UNG (hUNG) for which crystal structures are available in the DNA-free state, in complex with uracil-containing DNA and in complex with a DNA containing an abasic site [6–8, 10].

## Results and discussion

### Overall quality of the D4-DNA complex structure

The D4-DNA structure was refined to 2.89 Å resolution with R_cryst_ and R_free_ values of 21.6 and 26.5 %, respectively. The overall quality of the structural model is good as indicated by MolProbity scores [24] and map correlation coefficients (Table 2). The final model consists of two D4 subunits (chains A and B), a DNA double helix with 12 nucleotides in chain C and 10 in chain D, 4 glycerol (GOL) molecules of which one is in each active site and 118 solvent atoms. Glycerol molecules have been previously observed in the active site of D4 (*2owq, 2owr*) and *E. coli* UNG (*3eug*). Quality of electron density for residues 184–188 of the ‘Leu-intercalation’ loop was poor. Therefore, these five residues were not modeled. As a result, the discussion of the ‘Leu-intercalation’ loop for this structure is limited to interactions of loop residues 180–183. Also, side chain atoms of residues 189 and 190 (except for CB) are not included in the final model due to lack of electron density. These residues are located in a flexible area in crystal structures of D4 [13, 14].

**Table 2:** Data collection and refinement statistics for 4*qcb*

Wavelength [Å]	0.97918
Space group	P2_1_2_1_2_1_
Unit cell parameters [Å]	a = 39.40, b = 92.32, c = 142.88
*Data collection statistics*	
Resolution limit [Å]	56.50-2.89 (3.05-2.89)^a^
R_merge_^b,c^	0.158 (0.505)
R_meas_^b,c^	0.190 (0.609)
R_pim_^b,c^	0.105 (0.335)
Total number of observations	35850 (5327)
Total number unique	11991 (1750)
Mean I/σ (I)	6.1 (2.3)
CC_1/2_	0.986 (0.855)
CC*	0.996 (0.960)
Completeness [%]	97.9 (99.3)
Multiplicity	3.0 (3.0)
*Refinement statistics*	
Resolution range (Å)	56.50 - 2.89 (2.97 - 2.89)^a^
Number of unique reflections	11966 (886)
Completeness (%)	97.3 (99.1)
R_cryst_ (%)^d^	21.6 (29.7)
R_free_ (%)^d^	26.5 (32.6)
No. of protein residues	426
No. of DNA nucleotides	22
No. of GOL molecules	4
No. of water molecules	118
Wilson B-factor (Å^2^)	29.4
Average B-factors (Å^2^)	
Overall	31.4
Protein	28.5
DNA	56.5
GOL	36.6
Water	18.6
Coordinate error (maximum likelihood)	0.39
Correlation coefficient Fo-Fc	0.93
Correlation coefficient Fo-Fc free	0.88
Overall map CC (Fc, 2mFo-DFc)	0.82^e,f^
Ramachandran allowed (%)	99.5
Ramachandran disallowed (%)	0.5 [2 outliers, 1 in each subunit]
MolProbity clash score	8.7 [97^th^ percentile]
MolProbity score	1.9 [99^th^ percentile]

^a^Values in parentheses represent highest resolution shell

^b^R_meas_and R_pim_were calculated with SCALA [38] in the CCP4 program suite [39] using unmerged and not scaled data preprocessed by XDS [36, 37]. R_meas_is a merging R-factor independent of data redundancy while R_pim_ provides the precision of the averaged measurement, which improves with higher multiplicity [48] $$ \begin{array}{l}{\mathsf{R}}_{\mathsf{m}\mathrm{e}\mathsf{r}\mathsf{g}\mathrm{e}}={\displaystyle \sum_{\mathsf{hkl}}{\displaystyle \sum_{\mathsf{i}}\Big|{\mathsf{l}}_{\mathsf{i}}\left(\mathsf{h}\mathsf{k}\mathsf{l}\right)-<\mathsf{l}\left(\mathsf{h}\mathsf{k}\mathsf{l}\right)>\mathsf{l}/}}{\displaystyle \sum_{\mathsf{hkl}}{\displaystyle \sum_{\mathsf{i}}{\mathsf{l}}_{\mathsf{i}}\left(\mathsf{h}\mathsf{k}\mathsf{l}\right)}}\hfill \\ {}{\mathsf{R}}_{\mathsf{m}\mathrm{e}\mathsf{a}\mathsf{s}}={\displaystyle \sum_{\mathsf{hkl}}\sqrt{\mathit{\mathsf{N}}/\left(\mathit{\mathsf{N}}-\mathsf{1}\right)}{\displaystyle \sum_{\mathsf{i}}\Big|{\mathsf{l}}_{\mathsf{i}}\left(\mathsf{h}\mathsf{k}\mathsf{l}\right)-<\mathsf{l}\left(\mathsf{h}\mathsf{k}\mathsf{l}\right)>\mathsf{l}/}{\displaystyle \sum_{\mathsf{hkl}}{\displaystyle \sum_{\mathsf{i}}{\mathsf{l}}_{\mathsf{i}}\left(\mathsf{h}\mathsf{k}\mathsf{l}\right)}}}\hfill \\ {}{\mathsf{R}}_{\mathsf{pim}}={\displaystyle \sum_{\mathsf{hkl}}\sqrt{\mathsf{1}/\left(\mathit{\mathsf{N}}-\mathsf{1}\right)}{\displaystyle \sum_{\mathsf{i}}\Big|{\mathsf{l}}_{\mathsf{i}}\left(\mathsf{h}\mathsf{k}\mathsf{l}\right)-<\mathsf{l}\left(\mathsf{h}\mathsf{k}\mathsf{l}\right)>\mathsf{l}/}{\displaystyle \sum_{\mathsf{hkl}}{\displaystyle \sum_{\mathsf{i}}{\mathsf{l}}_{\mathsf{i}}\left(\mathsf{h}\mathsf{k}\mathsf{l}\right)}}}\hfill \end{array} $$

^c^R values for the low resolution shell of 56.50-9.14 Å are: R_merge_ 0.053; R_meas_ 0.065; R_pim_ 0.037

^d^The data included in the R_free_ set (5 %) were excluded from refinement

^e^Final R and R_free_ values based on map calculation in PHENIX [45, 46] are 20.8 % and 24.7 %, respectively

^f^Final R and R_free_ values based on comprehensive validation in PHENIX [45, 46] are 21.5 % and 26.8 %, respectively

**Fig. 2:**
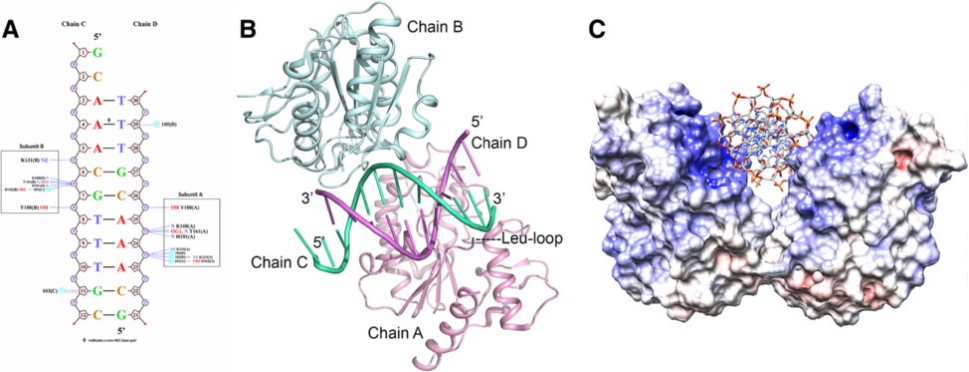
Structure of D4:DNA complex. **a**. Schematic diagram of the D4-DNA interactions. Bases are represented by one-letter codes (colored: A red, T blue, G green, C brown) and base pairs are connected by a solid black line (note that DA4 and DT29 form a non-WC base pair). The DNA backbone is drawn next to the bases (sugars as brown pentagons; phosphates as purple circles). DNA strand 1 (chain C) runs from top (5’) to bottom while the complementary strand 2 (chain D) runs in the opposite direction. Base numbers as in the PDB coordinate file are written inside the sugars. Interactions are plotted on either side of the strands; interacting protein residues are represented by their atom name (O in red, N in blue), residue name and number (chain identifier in parenthesis). Blue (dotted) lines represent hydrogen-bonded contacts (3.9 Å cut-off between ‘heavy’ atoms as defined in PDBePISA). Circles (in cyan) labeled ‘W’ indicate water-mediated interactions with DNA. Water molecules are labeled by their PDB number. Protein interactions of subunits A and B with DNA strands C and D are highlighted as boxes and labeled. NUCPLOT (http://www.ebi.ac.uk/thornton-srv/software/NUCPLOT/) [49] was used to generate the figure. **b**. DNA binding site in D4:DNA complex. Cartoon diagram illustrating the D4 chains A and B in light pink and light cyan and the corresponding DNA chains in pink (D) and bluish green (C) respectively. Location of the partially disordered ‘Leu-intercalation loop’ is labeled as Leu-loop and indicated with dotted lines. **c.** Molecular surface for D4:DNA complex. Molecular surface of the two D4 subunits is shown with the overlaid electrostatic potential contoured from negative (red) to positive (blue) [−10 kBT/e to +10 kBT/e]. The DNA helix is displayed as cartoon drawing (color: P yellow, O red, N blue, C grey). This figure was generated using UCSF Chimera (http://www.cgl.ucsf.edu/chimera/) [50]

In chain C all 12 nucleotides were modeled but only 10 could be modeled in chain D due to insufficient electron density. Of the 10 base pairs forming the DNA double helix, 9 show regular Watson-Crick (WC) base pairing interactions and one non-WC interaction (Fig. 2; Additional file 1: Table S2). In general electron density for the DNA is good for the central regions (C: nucleotides 5–8; D: nucleotides 23–26) that interact with protein residues as indicated by map correlation coefficients (0.92-0.96; average 0.94) and temperature factors (30.5-46.3 Å^2^; average 37.6 Å^2^). The overall quality of the electron density for the DNA double helix is displayed in Additional file 1: Figure S1.

A comparison of map correlation coefficients of the DNA nucleotides and the interface residues in D4 is presented in Additional file 1: Table S3. An electron density map (2mFo-DFc map contoured at 1.5σ level) for both D4-DNA interfaces (chains A and D; chains B and C) is displayed in Additional file 1: Figure S2.

As expected the DNA represents a B-form right-handed double helix. Puckering of the sugar ring for several nucleotides differs from the predominant C2’-endo conformation seen in B-DNA (Additional file 1: Table S4). Similar deviations in sugar puckering conformation have been observed in various protein-DNA complexes.

### Assembly of D4-DNA complex

DNA parameters derived from the analysis using the w3DNA web server (http://w3dna.rutgers.edu/) [25] are presented in Additional file 1: Tables S2 and S4. Analyses of DNA-DNA, protein-DNA and protein-protein interfaces using the PDBePISA web server (http://www.ebi.ac.uk/msd-srv/prot_int/pistart.html) [26] are shown in Table 3 and Additional file 1: Table S5.

**Table 3:** Detailed hydrogen bonding information for D4-DNA interactions in 4qcb (PISA analysis)

1. D4-DNA (chains A, D)	2. D4-DNA (chains B, C)
#1 D:DA24 [OP2] 2.69 A:LYS131 [NZ]	#1 C:DA5 [OP1] 3.76 B:THR130 [N]
#2 D:DA25 [OP1] 3.10 A:LYS160 [N]	#2 C:DC6 [OP2] 2.59 B:LYS131 [NZ]
#3 D:DA25 [OP2] 3.20 A:LYS160 [N]	#3 C:DG7 [OP1] 3.03 B:LYS160 [N]
#4 D:DA25 [OP2] 2.97 A:THR161 [N]	#4 C:DG7 [OP2] 3.22 B:LYS160 [N]
#5 D:DC26 [OP1] 3.13 A:TYR180 [OH]	#5 C:DG7 [OP2] 2.95 B:THR161 [N]
#6 D:DA25 [OP1] 2.88 A:HIS181 [N]	#6 C:DT8 [OP1] 2.76 B:TYR180 [OH]
	#7 C:DG7 [OP1] 2.91 B:HIS181 [N]
3. D4-DNA (chains A, C)	
#1 C:DC12 [OP1] 3.00 A:LYS87 [NZ]	

Conformations of the two D4 chains in the complex are also very similar with an *rmsd* value of 0.15 Å for superposition of all 213 residues in A and B. Each D4 chain binds only to one DNA strand. D4 chain A binds to DNA strand D and D4 chain B binds to DNA strand C (Figs. 2a, b). The electrostatic potential distribution shown in Fig. 2c illustrates the charge complementarity of the DNA binding surface of D4 and the negatively charged phosphate backbone of the DNA strands.

D4 chain B interacts with nucleotides 5–8 (ACGT) in the central part of chain C while D4 chain A interacts with nucleotides 23–26 (AAAC) of chain D. D4 residues at the protein-DNA interface are Ile67, Pro71, Gly128, Glu129, Thr130, Lys131, Gly159, Lys160, Thr161, Asp162, Tyr180, His181 and Ala183 (Table 3 and Additional file 1: Table S5). Polar protein-DNA interactions with the phosphate backbone involve hydrogen-bonds (2.6-3.2 Å) with Lys131, Lys160, Thr161, Tyr180 and His181 from each D4 chain (Table 3; Fig. 2). DNA binding residues in the two D4 chains superimpose very well (*rmsd* 0.13 Å).

Distortion in the DNA chains C and D such as bending and kinking are small compared to those observed in complexes of hUNG with specific uracil-containing DNA [6–8, 10]. The length of the DNA (~37-38 Å) is comparable to the value of 39–41 Å expected for an ideal extended B-form DNA; the widths of the major and minor groove are also similar to those for ideal B-form DNA.

Incidentally, the DNA double helix extends in the unit cell through non-covalent interactions between the ends of the DNA helices generated by the space group symmetry in a head-to-tail fashion (Additional file 1: Figure S3). Similar extended packing through DNA-DNA contacts of *ds*DNA with sticky or blunt ends (head-to-head and head-to-tail fashion) has been observed in other protein-DNA structures [27, 28]. On the other hand this arrangement of DNA chains may be a result of crystal packing.

Both protein-DNA interfaces in the complex have similar interface areas and interface residues (Additional file 1: Table S5). Interactions between the two D4 chains in the complex are minimal and confined to residues Glu32, Val33, Ser35, Trp36, Arg39, Ser132, Ile135, Try136 and Lys139. There is a salt bridge between residues Glu32 and Arg39 and a stacking interaction between Trp36 of two subunits.

### Conformational changes induced by DNA binding

**Fig. 3:**
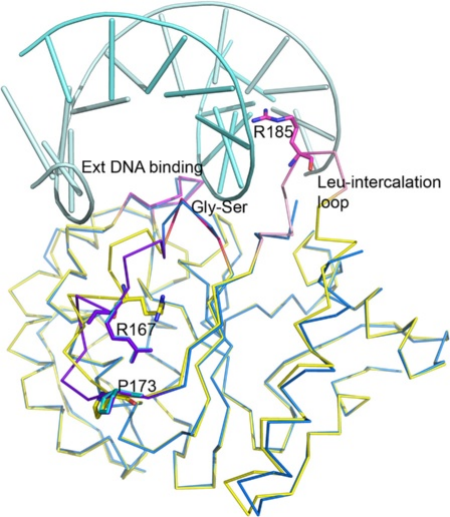
D4:DNA interactions. Comparison of the structures of D4 in free and DNA-bound states. Ribbon diagram showing superimposition of D4 subunits A and B (in marine and cyan) on D4 in the free form in yellow (*4dof*, chain A). The DNA is shown as cartoon (pale cyan) as bound to the A subunit in the complex. Areas important for DNA-interactions are highlighted on *4dof* chains in magenta (Extended DNA binding loop, residues 126–132), red (Gly-Ser loop, residues 159–162) and rose (Leu intercalation loop, residues 180–187). The Leu-intercalation loop is on *4dof*. Arg185 which replaces the conserved leucine residue is shown in stick model. The helix loop segment that shows large deviation (residues 164–174) is colored in violet. This region is critical for binding of A20 by D4. Residues Arg167 and Pro173 which play an important role in binding of A20 are shown as stick models and labeled

Upon formation of productive DNA complexes, UNGs undergo an ‘open to close’ conformational transition [6–10]. Such a conformational transition is not expected in the present complex since the DNA in the complex does not contain uracil. D4 residues involved in binding DNA are localized in three structural areas: the extended DNA-binding loop (residues 126–132), the Gly-Ser loop (residues 159–162) and the Leu-intercalation loop (residues 180–187). These three regions are labeled in Fig. 3. Involvement of these sites in DNA-binding and the resulting structural changes in the protein are discussed below. Largest deviations in the DNA complex are noticed in the extended helix/loop segment (residues 164–174,s1 site) joining the two C-terminal strands of the central β-sheet, and in the Leu-intercalation loop (s2 site). These two regions show varying degrees of disorder in D4 structures and portions of these areas are missing from several final models. Therefore, we examined the electron density maps for D4 structures especially focusing on these areas. In the following discussion we used reference free-D4 structures in which completeness of the model in relevant regions of the protein was good and supported by electron density. We also used the structure of the D4:uracil complex (*4lzb*) for comparison [14]. This structure contains 12 molecules in the asymmetric unit and the s1 and s2 sites could be modeled in 11.

### Extended DNA binding loop (residues 126–132)

**Fig. 4:**
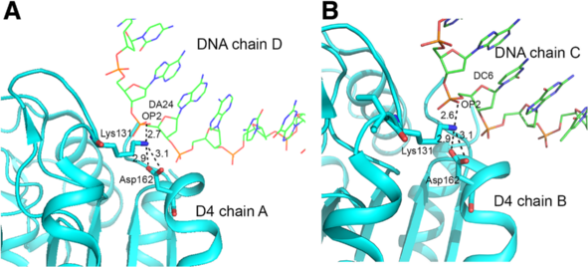
Protein-DNA interactions at the extended DNA-binding site. **a** and **b** shown in left and right panel are hydrogen bonding interactions (identified by PISA analysis) involving D4 chains A and B, respectively. Relevant D4 residues are shown in stick models and are labeled. DNA chains are shown as lines. Hydrogen bonding distances shown are in a. Interaction of Lys131 side chain NZ atom with Asp162 carboxyl group may be important for local conformational stability. Electron density for this region of DNA complex is shown in Additional file 1: Figure S4

In the DNA complex the side chain amino group of Lys131 of each D4 subunit forms a strong hydrogen bond (2.6 and 2.7 Å) with a phosphate oxygen atom of the partner DNA strand. However, there is no movement in this region of D4 upon DNA binding. Main chain and side chain atoms of Lys131 and neighboring residues superimpose very well with corresponding atoms in free-D4 structures *4dof, 4dog* and *4lzb*. In all structures the Lys131 side chain extends towards the spatially close Gly-Ser loop and engages in hydrogen bonding with the carboxyl oxygen atoms of Asp162. As a result, the extended DNA-binding loop tilts slightly towards the Gly-Ser loop (Fig. 4). Quality of electron density for Lys131, Asp162 and areas adjacent to these residues is usually very good in these crystal structures (Additional file 1: Figure S4). In a previously published study, the Lys131Val mutant was found to be defective in processivity function [22]. Since this mutant did not show glycosylase activity it was assumed to have a major conformational defect [22]. Both Lys131 and Asp162 are strictly conserved in UNGs of the orthopoxviruses [15] and thus may play an important role in specialized functions of these viral UNGs, such as a component of the processivity factor. Since neither of these residues is conserved in other UNGs they are less likely to play any direct role in catalysis. On the other hand interactions of Lys131 with Asp162 on the spatially adjacent helix/loop may be important for the stability of the local structure. Therefore the loss of glycosylase activity in the valine mutant may be due to structural impairment. At the same time, D4 surface near Asp162 is critical for its interactions with A20 [22] and therefore, structural changes due to the loss of interactions between Lys131 and Asp162 may affect the processivity function.

In a study combining H/D exchange mass spectroscopy and computational docking Roberts et al. [29] identified two extended non-sequence-specific DNA-binding surfaces in hUNG (residues 210–220 and 251–264) that did not show contacts with DNA in the crystal structures. In D4 the corresponding regions are residues 126–136 and 165–178. The latter segment in D4 is engaged in homodimer formation in the absence of A20 and in heterodimer formation in the presence of A20 [20]. Thus this (residue 165–178) may not represent a potential DNA-binding site in D4.

### Gly-Ser loop (residues 159–162)

**Fig. 5:**
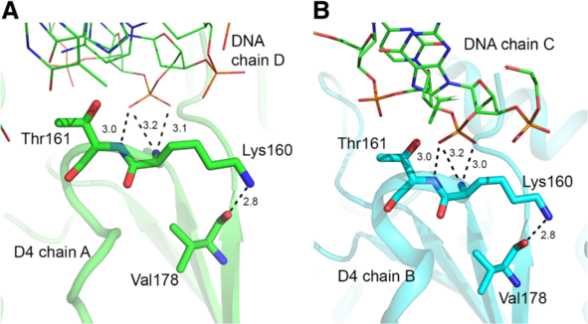
Protein-DNA interactions at the Gly-Ser loop. Hydrogen bonding interactions between the D4 residues in A and B subunits with partner DNA strands are shown in left and right panel. Relevant D4 residues are shown in stick models and are labeled. DNA chains are shown as lines. Hydrogen bonding distances shown are in Å. Also NZ atom of Lys160 forms hydrogen bonding interactions with the peptide oxygen atom of Val178 located on the C-terminal strand of the central β-sheet

The s2 site encompasses the Gly-Ser loop. This region shows varying degrees of flexibility in different D4 structures but could be modeled in both subunits in the DNA complex. Quality of electron density in this area of D4 was sufficient for unambiguously placing main chain and side chain atoms of all residues. For comparison of this site we selected the free-D4 structure *4dof* (chains A, B) and the D4:uracil complex (*4lzb,* chain D, F). Comparison of the s2 site in the DNA-complex with free-D4 structures reveals a slight reorganization in this area of the molecule (Fig. 5). Two amino acid residues, Lys160 and Thr161, are involved in hydrophilic interactions with the DNA. Main chain nitrogen atom of Lys160 interacts with two oxygen atoms on one phosphate group (Table 3). Thr161 hydroxyl oxygen atom is hydrogen bonded to one of the oxygen atoms of the same phosphate also through the peptide nitrogen atom. Notably, Lys160 and Thr161 are unique residues in poxvirus UNGs and are replaced by glycine and serine respectively in both *E. coli* and human enzymes. We showed that D4 mutant Lys160Val was deficient in processivity function but retained catalytic activity and DNA binding ability [22]. In all D4 structures, side chain of Lys160 extends toward the neighboring β-strand and its NZ atom forms a hydrogen bond with the peptide oxygen atom of Val178 on this strand. Loss of this contact in the Lys160Val mutant may impact the structure of this area of D4. Notably, a hydrophobic pocket at this site has been shown to be important for D4’s interaction with A20 [20].

### Leu-intercalation loop (residues 180–187)

**Fig. 6:**
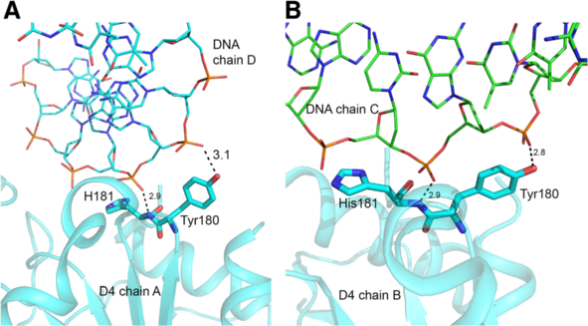
Protein-DNA interactions at the Leu-intercalation loop. Cartoon and stick drawing displaying hydrogen bonding interactions at the Leu-intercalation loop region between the D4 residues in A and B subunits with partner DNA strands. Relevant D4 residues are shown in stick models and are labeled. DNA chains are shown as lines. Hydrogen bonding distances shown are in Å. Residues 184–188 were missing from both subunits

The s3 area, which includes the ‘Leu-intercalation loop’, is important for the catalytic mechanism. Electron density for this segment of D4 structure in *4dog* and *3 nt7* was good [22]. In the DNA-complex quality of electron density for this loop was inadequate for modeling the entire loop. Therefore, residues 184–188 were omitted from the final model. Two amino acid residues immediately preceding the disordered region of D4, namely Tyr180 and His181, directly interact with DNA. Side chain hydroxyl group of Tyr180 and the main chain nitrogen atom of His181 form contact with oxygen atoms of two phosphate groups from the DNA (Fig. 6). Tyr180 is conserved in UNGs of poxviruses but not in other UNGs. His181 is a critical catalytic residue and is conserved in UNGs across the species. In *4zlb* we noticed some deviations in the position of His181 in different subunits. Generally, in a productive uracil complex, the distance between the NE2 atom of histidine and the O2 atom of uracil is significantly shorter than in non-productive complexes [6–8]. In *4lzb*, the distance between O2 atom of uracil and His181 NE2 atom varied from 2.8-4.8 Å in different subunits [14]. In the DNA complex His181 side chain moved farther away from the active site area. The missing segment includes Arg185, which is equivalent to the conserved leucine residue (272 in hUNG) of the Leu-intercalation loop. In the mutational analysis mentioned above D4 Arg187Val mutant was also shown to be unable to support processive DNA synthesis but retained binding to A20 and DNA [22]. Unfortunately, due to disorder in this loop detailed comparison of this functionally significant region is not possible from the present structure.

### Movement of Arg167 and implications for A20 binding

**Fig. 7:**
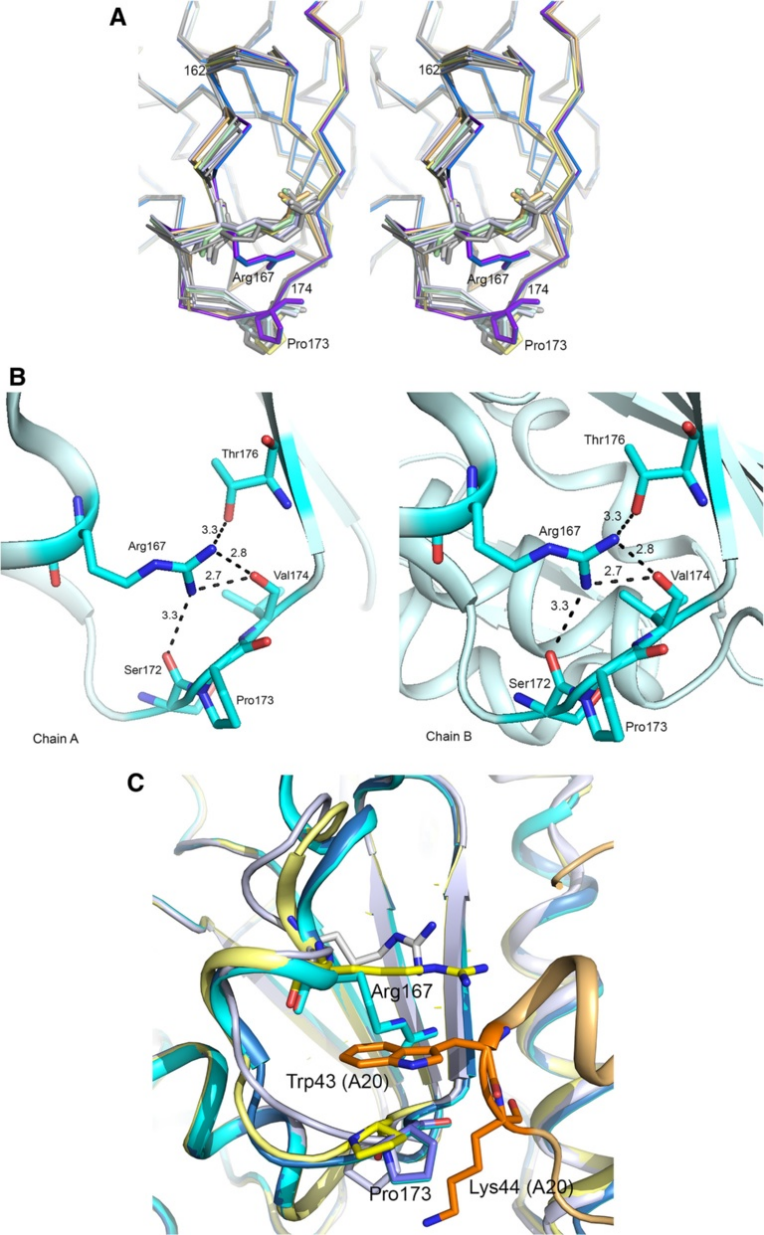
Movement of Arg167 in the D4:DNA complex and A20 binding. **a**. Arg167 side chain of D4 in the DNA complex and in DNA-free D4. Stereo diagram showing movement of Arg167 and Pro173 residues of D4 chains A and B in the DNA complex (shown in blue and purple violet colors) superimposed on D4 subunits in different subunits of the uracil complex (*4lzb*) shown in various shades of yellow and green. Arg167 and Pro173 residues are shown in stick model. **b**. Hydrogen bonding interactions of Arg167 in the DNA complex. Close up view of the above region showing hydrogen bonding interactions of Arg167 of the two D4 chains A and B of the DNA complex in the left and right panel, respectively. **c**. Orientations of Arg167 and Pro173 in the DNA complex. Cartoon drawing showing close up view of the A20 binding site on D4. D4 subunits from free-D4 (*4dof* subunit A, white), and D4 subunits A and B from the DNA complex (cyan and marine) are superimposed on the D4 subunit of the D4:A20 complex (*4od8*, yellow). The A20 molecule in *4od8* is colored in wheat. Trp43 and Lys44 residues of A20 are shown in stick model (carbon atoms in orange). Arg167 residues of D4 chains are also shown in stick models and carbon atoms of each stick model are colored according to the chain. Orientations of Arg167 and Pro173 of the D4 subunits in the DNA complex are unfavorable for binding of A20 in the groove

Most significant changes in the DNA complex were noticed in residues Arg167 and Pro173 although neither of these residues is involved in direct interaction with DNA. In *4dof* and *4lzb*, orientation of the Arg167 side chain is stabilized by a strong hydrogen bond between its NH2 atom and the main oxygen atom of Thr176. In both D4 subunits in the DNA complex, Arg167 side chain is directed away from Thr176 (Fig. 7a). The Arg167 side chain is oriented such that nitrogen atoms of the terminal amino groups form hydrogen bonds with the peptide oxygen atom of Val174. Position of Arg167 side chain is further stabilized by polar interactions of its amino groups with the hydroxyl oxygen atom of Thr176 side chain on one side and the main chain oxygen atom of Ser172 on the other (Fig. 7b).

In the crystal structure of the D4:A20 complex, the aromatic side chain of Trp43 of A20 occupies a hydrophobic pocket referred to as groove by Contesto-Richefeu et al. [22] and inserts into the space between Pro173 and Arg167 side chain. Movement of Arg167 side chain and Pro173 in the A20 complex (as compared to free D4 structures) favors this interaction. On the other hand in the DNA-complex, orientation of Arg167 side chain would interfere with binding of A20 at this site. Interestingly, movement of Pro173 in the DNA-complex also seems to create an unfavorable steric environment for binding of A20. While in the D4:A20 complex, Pro173 of D4 moves in a direction that allowed packing of Lys44 side chain of A20, in the DNA complex the carbonyl oxygen Pro173 would be in close contact (~1.5 Å) with the CB atom of Lys44 of A20 (Fig. 7c). Thus the structure of the present DNA complex suggests that DNA binding induces conformational changes in D4 that may interfere with its binding to A20. While D4 can independently bind both DNA and A20, perhaps in the formation of the heterotrimeric complex, D4-A20-interaction precedes the DNA-binding event.

### Comparison of hUNG and D4

**Fig. 8:**
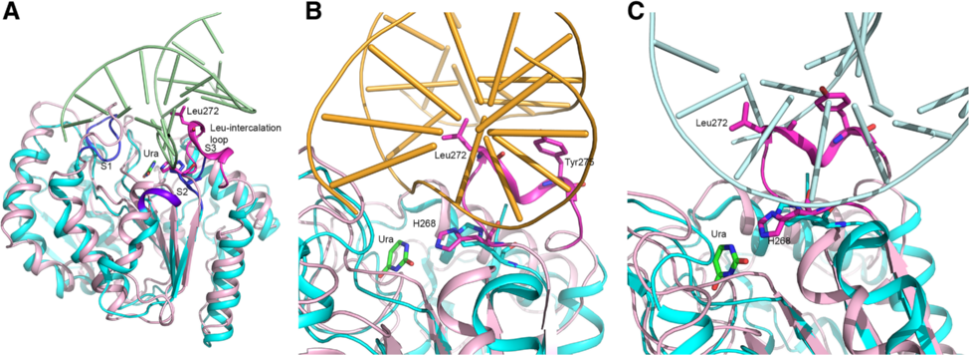
Comparison of D4:DNA complex with hUNG:DNA complex. **a**. Overall comparison. Structure of the A subunit of the D4:DNA complex (cyan) is superimposed on the structure of hUNG:DNA (*1ssp*, light pink). DNA chain from 1*ssp* is shown in cartoon drawing (pale green). Uracil molecule cleaved and bound at the uracil binding pocket in hUNG is shown in stick model (carbon in green). His181 of D4 and the corresponding His268 of hUNG are shown in stick models. Leu272 of the Leu-intercalation loop (magenta) in hUNG is also shown in magenta sticks. DNA-binding areas on D4 are colored darker: S1 site, residues 126–132, S2 site residues 159–162 and S3 site residues 180–187. The corresponding residue of Leu272 in D4 is disordered. **b**. Close up view 1. Close up view showing superimposition of D4 subunit A of D4:DNA complex (cyan) on hUNG:DNA complex structure (light magenta). DNA bound to hUNG is shown in cartoon (light orange). Uracil molecule (Ura) in hUNG uracil binding pocket is shown as stick model (carbon in green). Catalytic His268 of hUNG and the corresponding H181 of D4 are also shown in stick model. **c**. Close up view 2. Cartoon diagram shows superimposition of the hUNG chain (light magenta) from the DNA complex *1ssp* on the D4 chain A (cyan) of the D4:DNA complex. DNA shown in cartoon model is from the D4:DNA complex (chain D, light cyan). Unless specified otherwise figures were generated using PyMOL (http://pymol.org/) [51]

Crystal structures of hUNG have been reported in the free-enzyme form [*1akz*; 6] and in complex with damaged DNA containing uracil [*1ssp*; 6]. Structure of mutant hUNG in which the leucine residue (272) of the Leu-intercalation loop was altered to alanine was described in complex with DNA containing an abasic site [*2ssp*; 6]. Superimposition and comparison of the structure of hUNG with D4 in their DNA complexes demonstrate the divergences and general overlap in the areas important for DNA binding and UNG catalytic mechanism. The three DNA binding sites described above are shown in Fig. 8. Structural comparison of hUNG in the free-state and in complex with different DNA constructs demonstrated that major conformational changes in UNGs are promoted by the steps involved in generating and stabilizing the flipped out nucleotide upon recognition of uracil [10]. Upon binding an undamaged DNA, these conformational changes are not expected in D4. Superimposition of the structure of the D4:DNA complex and the DNA complex of hUNG (*1ssp*) reveals that the Gly-Ser loop and the Leu-intercalation loop provide the main interactions with DNA. Part of the Leu-intercalation loop is disordered in the D4:DNA complex. In hUNG, the catalytic histidine (His268 in *1ssp*) clearly moves slightly closer towards the uracil binding pocket as compared to the D4:DNA complex. It should be noted that in DNA-free state this histidine residue superimposes well in D4 and hUNG (see Fig. 8). The interactions between the DNA and D4 at the extended DNA-binding site may be facilitated because of the larger size of the bound DNA as compared to hUNG:DNA complexes.

## Conclusions

The D4-DNA complex described here provides the structural framework for recognizing the DNA binding residues and motifs in poxvirus UNGs. The process of sequence-specific recognition and binding of DNA includes non-specific interaction as an early or intermediate step [30–33]. The structure with undamaged *ds*DNA may represent a snapshot of an early DNA-protein interaction in preparation for the recognition complex for UNG [34]. It remains to be seen if the Leu-intercalation loop transitions from its disordered state to an ordered state upon recognition of a damage in the DNA. Some of the unique structural features of D4 have evolved for undertaking its novel role in viral replication. Structure presented here provides potential understanding of DNA-binding by D4 in the poxvirus replication machinery.

## Methods

### Preparation and crystallization of D4-DNA complex

Custom DNA oligos purified by standard desalting method were ordered from Integrated DNA Technologies (IDT). The 12mer DNA oligo was designed to be self-complementary: 5’-GCA AAC GTT TGC-3’. The DNA oligo was dissolved in annealing buffer (10 mM Tris, pH 7.5, 50 mM NaCl, 1 mM EDTA) at a concentration of 5 mM. Annealing using a Perkin Elmer GeneAmp PCR System 2400 resulted in a blunt-end 12mer double-stranded DNA construct with 12 base pairs (final concentration 2.5 mM):

- 5’-GCA AAC GTT TGC-3’ (forward strand)
- 3’-CGT TTG CAA ACG-5’ (reverse complementary strand)

The following annealing protocol was used:

1. Heating to 368 K in 5 min;
2. Cooling to 338 K in 5 min;
3. Cooling to 328 K in 5 min;
4. Cooling to 318 K in 5 min;
5. Cooling to 298 K in 5 min;
6. Cooling to 277 K in 40 min.

DNA oligos and annealed *ds*DNA were kept at 253 K until use.

Tag-free D4 (tfD4) was purified as previously described [14, 35]. The purified protein contained three vector encoded residues (−GSH) after the hexa-histidine tag was cleaved off.

Protein solution at 3 mg/mL (~120 μM in 25 mM HEPES buffer, pH 7.3, 60 mM KCl, 1 mM TCEP) was incubated with previously annealed self-complementary non-specific *ds*DNA in a 1:1.2 molar ratio at 277 K for 1 h. High-throughput crystallization screening using the PEGs and Protein Complex Suites from Qiagen was conducted on a Crystal Gryphon (Art Robbins Instruments). Thin plate-like crystals were obtained in hanging drop vapor diffusion at 293 K. The drops contained 1 μL protein plus 0.5 μL reservoir solution containing 16 % PEG6K, 0.08 M TRIS buffer, pH 8.5, 20 % glycerol. For cryo-freezing, crystals were directly plunged into liquid nitrogen.

### Data collection and refinement

Intensity data were collected on a Pilatus 6 M detector at the Advanced Photon Source on NE-CAT beamline 24-ID-C. Data were processed with XDS [36, 37] and SCALA [38] of the CCP4 program suite [39] as part of the RAPD data-collection strategy at NE-CAT (https://chem.cornell.edu/rapd/). A total of 120 images (1° width) were collected. While the first 75 frames showed overall good statistics frames 76–120 showed increased mosaicity and R_merge_ values. The processed data from frames 1–75 were indexed in the orthorhombic crystal system. Probabilities for Laue group P222 and space group P2_1_2_1_2_1_ based on systematic absences were 0.856 and 0.967, respectively. Diffraction data extending to 2.89 Å resolution were used for molecular replacement and refinement. CC_1/2_ and CC* values at 2.89 Å resolution were 0.86 and 0.96, respectively [40, 41]. Data-collection statistics are listed in Table 1.

The unit-cell parameters (a = 39.4 Å, b = 92.3 Å, c = 142.9 Å; space group P2_1_2_1_2_1_) suggested two protein subunits in the asymmetric unit (52 % solvent; Matthews coefficient V_M_ = 2.6). The crystal structure was solved by molecular replacement with Phaser (version 2.5.6) [42] using the coordinates of one subunit from the native D4 structure (*4dof*) as search model. The observed pseudo-translation vector was used to position the second subunit in the asymmetric unit. Of the alternative space groups in Laue group P222 only space group P2_1_2_1_2_1_ provided solutions for the translation function. The R value of the refined solution was 45.2 % (RFZ = 5.8, TFZ = 9.3, LLG = 1803). After 1 round of refinement (10 cycles) of the protein coordinates (R_cryst_ and R_free_ dropped to 29.2 % and 32.9 %, respectively) in REFMAC (version 5.8.0073) [43] electron density for the DNA double helix became visible in sigmaA weighted 2mF_o_-DF_c_ and mF_o_-DF_c_ difference electron density maps (m is the figure of merit and D is the sigmaA weighting factor). Using modeling tools in Coot (version 0.8-pre) [44] B-form DNA nucleotides were fitted into the electron density maps. The forward strand (chain C) of the *ds*DNA is numbered from 1–12 (5’ to 3’) while the reverse complementary strand (chain D) is numbered 21–32 (5’ to 3’). Base pairing occurs between the base of nucleotide 1 and the base of nucleotide 32 and similarly between the nucleotide 12 and 21 (and so forth). For structure refinement we used automatically generated local non-crystallographic symmetry (NCS) restraints in REFMAC. Structure validation was accomplished using PHENIX (version 1.9-1692) [45, 46], MolProbity [24], QualityCheck (http://smb.slac.stanford.edu/jcsg/QC/) [47], and the new wwPDB X-ray validation server (http://wwpdb-validation.wwpdb.org/validservice/). PHENIX and REFMAC were used for final map calculations. Refinement statistics are listed in Table 1. The structure has been deposited in PDB under *4qcb*. The difference between R and R_free_, although within reasonable limit for structures in similar resolution, is somewhat higher and may result from incompleteness of the models (for DNA and protein).

## Additional files

Additional file 1: Table S1. Sequence alignment of six specific regions in UNG enzymes that include the motifs for DNA binding and catalysis. **Table S2.** Detailed hydrogen bonding information for DNA base pairing (w3DNA analysis) in 4QCB. **Table S3.** Map correlation coefficients and average B values for protein interface residues and DNA nucleotides. **Table S4.** Conformational sugar parameters in 4QCB (w3DNA analysis). **Table S5.** Analysis of protein-protein, protein-DNA and DNA-DNA interfaces in 4*qcb* (PISA analysis). **Figure S1.** SigmaA weighted 2mFo-DFc and mFo-DFc omit maps for DNA region. DNA double helix with chains C and D. A. 2mFo-DFc map (contoured at 1.5σ level). B. mFo-DFc omit map (contoured at 2.5σ level). C. 2mFo-DFc simulated annealing omit map (contoured at 1.0σ level). **Figure S2.** SigmaA weighted 2mFo-DFc map for D4-DNA interfaces. A. Stick model representation of D4-DNA interface 1 (chains A and D). B. 2mFo-DFc map of D4-DNA interface 1 (chains A and D). C. Stick model representation of D4-DNA interface 2 (chains B and C). D. 2mFo-DFc map of D4-DNA interface 2 (chains B and C). **Figure S3.** Packing of D4-DNA complex. **Figure S4.** Electron density map.

## Abbreviations

D4: Vaccinia uracil-DNA glycosylase
BER: Base excision repair
AP: Apurinic/apyrimidinic
Cyt: Cytosine
Ura: Uracil
UDG: Uracil-DNA glycosylase
UNG: Family I uracil-DNA glycosylase
*ss*DNA: Single-stranded DNA
*ds*DNA: Double-stranded DNA
hUNG: Human UNG
GOL: glycerol
EDTA: Ethylenediaminetetraacetic acid
PEG: Polyethylene glycol

## Footnotes

1 PDBID’s are shown in italics

2 Uracil in this complex is not a product of glycosylase activity
