# Supplementary material for: Binding of undamaged double stranded DNA to vaccinia virus uracil-DNA Glycosylase
Source: BMC Struct Biol. 2015 Jun 2;15:10. doi: 10.1186/s12900-015-0037-1 (PMC4450493; doi:10.1186/s12900-015-0037-1)
Supplement: Additional file 1: Table S1. — Sequence alignment of six specific regions in UNG enzymes that include the motifs for DNA binding and catalysis. Table S2. Detailed hydrogen bonding information for DNA base pairing (w3DNA analysis) in 4QCB. Table S3. Map correlation coefficients and average B values for protein interface residues and DNA nucleotides. Table S4. Conformational sugar parameters in 4QCB (w3DNA analysis). Table S5. Analysis of protein-protein, protein-DNA and DNA-DNA interfaces in 4qcb (PISA analysis). Figure S1. SigmaA weighted 2mFo-DFc and mFo-DFc omit maps for DNA region. DNA double helix with chains C and D. A. 2mFo-DFc map (contoured at 1.5σ level). B. mFo-DFc omit map (contoured at 2.5σ level). C. 2mFo-DFc simulated annealing omit map (contoured at 1.0σ level). Figure S2. SigmaA weighted 2mFo-DFc map for D4-DNA interfaces. A. Stick model representation of D4-DNA interface 1 (chains A and D). B. 2mFo-DFc map of D4-DNA interface 1 (chains A and D). C. Stick model representation of D4-DNA interface 2 (chains B and C). D. 2mFo-DFc map of D4-DNA interface 2 (chains B and C). Figure S3. Packing of D4-DNA complex. Figure S4. Electron density map. [file 12900_2015_37_MOESM1_ESM.docx]

**Binding of undamaged double stranded DNA to Vaccinia Virus Uracil-DNA Glycosylase**

**N. Schormann,^1^ S. Banerjee,^2^ R. Ricciardi^3^ and D. Chattopadhyay^1^***

^1^Department of Medicine, University of Alabama at Birmingham, Birmingham, AL 35294, USA

^2^Northeastern Collaborative Access Team and Department of Chemistry and Chemical Biology, Cornell University, Argonne, IL 60439, USA

^3^Department of Microbiology, School of Dental Medicine, Abramson Cancer Center, University of Pennsylvania, Philadelphia, PA 19104, USA

*Correspondence: [debasish@uab.edu](mailto:debasish@uab.edu) (Debasish Chattopadhyay)

[nschorm@uab.edu](mailto:nschorm@uab.edu) (Norbert Schormann)

[sbanerjee@anl.gov](mailto:sbanerjee@anl.gov) (Surajit Banerjee)

[ricciard@upenn.edu](mailto:ricciard@upenn.edu) (Robert Ricciardi)

**Supplementary Information: Tables and Figures**

**Tables S1-S5**

**Figure S1-S4**

**Table S1. Sequence alignment of six specific regions in UNG enzymes that include the motifs for DNA binding and catalysis.**

Region 1 Region 2 Region 3 Region 4

Human 143-GQDPYH-148 165-PPPPS-169 201-LLLN-204 210-RAHQANS-216

*E. coli* 62-GQDPYH-68 84-AIPPS-88 120-LLLN-123 129-RAGQAHS-135

HSV-1 86-GQDPYH-91 108-PPPPS-112 143-LLLN-147 153-KRGAAAS-159

VACV 66-GIDPYP-71 84-FTK**K**S-88 117-IPWN-120 126-KLGET**K**S-132

VARV 66-GIDPYP-71 84-FTKKS-88 117-IPWN-120 126-KLGETKS-132

MPXV 66-GIDPYP-71 84-FTKKS-88 117-IPWN-120 126-KLGETKS-132

CPXV 66-GIDPYP-71 84-FTKKS-88 117-IPWN-120 126-KLGETKS-132

CMPV 66-GIDPYP-71 84-FTKKS-88 117-IPWN-120 126-KLGETKS-132

SPPV 66-GIDPYP-71 84-FSKKT-88 117-FPWN-120 126-KIGETKS-132

SWPV 66-GIDPYP-71 84-FSKKT-88 117-IPWN-120 126-KVGETKS-132

Region 5 Region 6

Human 245-WGSY-248 267--AHPSPLSVYR-276

*E. coli* 164-WGSH-167 186--PHPSPLSAHR-195

HSV-1 188-WGTH-191 208-FSHPSPLS--K-216

VACV 159-G**KTD**-162 180-**Y**-**H**PAA**R**--D**R**-187

VARV 159-GKTD-162 180-Y-HPAAR--DR-187

MPXV 159-GKTD-162 180-Y-HPAAR--DH-187

CPXV 159-GKTD-162 180-Y-HPAAR--DR-187

CMPV 159-GKTD-162 180-Y-HPAAR--DR-187

SPPV 159-GKTD-162 180-Y-HPAAR--DR-187

SWPV 159-GKTD-162 180-Y-HPAAR--DK-187

Highlighted in the VACV sequence (based on our D4-DNA structure) are:

1. DNA interface residues with hydrogen bonds (bold)
2. DNA interface residues with non-bonded contacts (shaded)

Motifs included in regions 1-6 are:

1. Region 1: Catalytic water-activating loop
2. Region 2: Pro-rich loop
3. Region 3: Uracil specificity β-strand
4. Region 4: Extended DNA binding loop
5. Region 5: Gly-Ser loop
6. Region 6: Leu-intercalation loop

HSV-1: Herpes Simplex virus 1

VACV: Vaccinia virus

VARV: variola major virus

MPXV: monkeypox virus

CPXV: cowpox virus

CMPV: camelpox virus

SPPV: sheeppox virus

SWPV: swinepox virus

**Table S2. Detailed hydrogen bonding information for DNA base pairing (w3DNA analysis) in *4QCB*.**

1 A-----T N1 – N3 2.85 N6 – O4 2.94

2* A-----T N7 – N3 3.32 N6 – O2 2.40

3 A-----T N1 – N3 2.97 N6 – O4 2.71

4 C-----G N3 – N1 2.91 N4 – O6 3.03 O2 – N2 2.69

5 G-----C N1 – N3 2.93 O6 – N4 3.48 N2 – O2 2.35

6 T-----A N3 – N1 2.66 O4 – N6 2.72

7 T-----A N3 – N1 2.52 O4 – N6 2.41

8 T-----A N3 – N1 2.96 O4 – N6 2.81

9 G-----C N1 – N3 2.42 O6 – N4 2.52 N2 – O2 2.41

10 C-----G N3 – N1 3.14 N4 – O6 2.99 O2 – N2 3.23

*Note: Base pair 2 (A---T) shows a non-Watson-Crick base pair. Distances are in Å.

**Table S3. Map correlation coefficients and average B values for protein interface residues and DNA nucleotides.**

DNA interface nucleotides, chain C:

1(DG) 2(DC) 3(DA) 4(DA) 5(DA) 6(DC) 7(DG) 8(DT) 9(DT) 10(DT) 11(DG) 12(DC)

CC 0.65 0.67 0.79 0.81 0.93 0.96 0.96 0.92 0.87 0.86 0.80 0.79

B_factor_ 64.4 72.1 75.9 81.9 46.3 30.8 37.7 39.5 54.8 75.6 71.9 60.9

DNA interface nucleotides, chain D:

21(DG) 22(DC) 23(DA) 24(DA) 25(DA) 26(DC) 27(DG) 28(DT) 29(DT) 30(DT)

CC 0.78 0.85 0.93 0.96 0.96 0.93 0.93 0.86 0.83 0.78

B_factor_ 53.9 70.0 43.5 34.4 30.5 38.3 48.2 76.5 74.3 63.6

D4 interface residues, subunit A:

I67 P71 G128 E129 T130 K131 G159 K160 T161 D162 Y180 H181 A183

CC 0.97 0.96 0.95 0.93 0.95 0.95 0.97 0.94 0.94 0.96 0.94 0.96 0.89

B_factor_ 23.2 23.7 26.8 29.7 26.2 24.1 28.4 28.4 25.6 25.1 34.6 31.5 38.7

D4 interface residues, subunit B:

I67 P71 G128 E129 T130 K131 G159 K160 T161 D162 Y180 H181 A183

CC 0.96 0.97 0.96 0.91 0.94 0.94 0.97 0.94 0.95 0.95 0.96 0.96 0.90

B_factor_ 23.0 22.9 30.3 37.4 30.0 31.1 28.4 29.6 27.4 24.8 27.0 30.5 39.3

DNA interface nucleotides are highlighted by shading.

**Table S4. Conformational sugar parameters in *4QCB* (w3DNA analysis).**

Strand I (chain C) Strand II (chain D)

Base ID tm P Puckering Base ID tm P Puckering

1 A C3 24.7 179.0 C2'-endo 1 T D30 32.7 144.4 C2’-endo

2 A C4 45.7 137.5 C1'-exo 2 T D29 37.3 71.9 C4'-exo

3 A C5 36.3 163.4 C2'-endo 3 T D28 37.8 180.3 C3'-exo

4 C C6 32.5 156.5 C2'-endo 4 G D27 42.9 150.6 C2'-endo

5 G C7 33.9 175.1 C2'-endo 5 C D26 35.1 152.5 C2'-endo

6 T C8 39.2 145.3 C2'-endo 6 A D25 30.8 173.9 C2'-endo

7 T C9 35.1 138.2 C1'-exo 7 A D24 36.6 165.4 C2'-endo

8 T C10 46.1 152.7 C2'-endo 8 A D23 35.2 160.2 C2'-endo

9 G C11 42.2 142.0 C1'-exo 9 C D22 20.1 195.0 C3'-exo

10 C C12 31.5 127.3 C1'-exo 10 G D21 35.5 128.3 C1'-exo

tm: amplitude of pseudorotation of the sugar ring

P: phase angle of pseudorotation of the sugar ring

**Table S5. Analysis of protein-protein, protein-DNA and DNA-DNA interfaces in *4QCB* (PISA analysis).**

Interface residues^a^ Interface area [Å^2^]

1. DNA (*CSS 1.00; 26 H-bonds)

DNA strand 1 (chain C) 11 490 (17% of total)

DNA strand 2 (chain D) 10 475 (20% of total)

1. D4-DNA (CSS 0.64; 6 H-bonds)

D4 (chain A) 13^b^ 271 (3% of total)

DNA strand 2 (chain D) 4 [23-26] 314 (13% of total)

1. D4-DNA (CSS 0.85; 7 H-bonds)

D4 (chain B) 13^c^ 272 (3% of total)

DNA strand 1 (chain C) 4 [5-8] 319 (11% of total)

1. D4 (CSS 0.06; 2 H-bonds, 2 salt bridges)

D4 (chain A) 9^d^ 308 (3% of total)

D4 (chain B) 8^e^ 308 (3% of total)

E. D4-DNA (CSS 0.00; 1 H-bond)

D4 (chain A) 3^f^ 110 (1% of total)

DNA strand 1 (chain C) 4 [3-4, 11-12] 108 (4% of total)

F. D4-DNA (CSS 0.04; 0 H-bond)

D4 (chain B) 3^g^ 101 (1% of total)

DNA strand 1 (chain D) 4 [21-22, 29-30] 107 (5% of total)

*CSS stands for the Complexation Significance Score, which indicates how significant for assembly formation the interface is.

^a^Interface residues are defined as residues that bury at least part of their solvent accessible surface upon binding.

^b^Protein-DNA interface residues in A: Ile67, Pro71, Gly128, Glu129, Thr130, Lys131, Gly159, Lys160, Thr161, Asp162, Tyr180, His181, Ala183

^c^Protein-DNA interface residues in B: Ile67, Pro71, Gly128, Glu129, Thr130, Lys131, Gly159, Lys160, Thr161, Asp162, Tyr180, His181, Ala183

^d^Protein-protein interface residues in A: Glu32, Val33, Ser35, Trp36, Arg39, Ser132, Ile135, Tyr136, Lys139

^e^Protein-protein interface residues in B: Glu32, Val33, Trp36, Arg39, Ser132, Ile135, Tyr136, Lys139

^f^Protein-DNA interface residues in A: Lys87, Asn165, Ala183

^g^Protein-DNA interface residues in B: Lys87, Asn165, Ala183

Listed in brackets are the interacting nucleotides.

**Figure S1. SigmaA weighted 2mFo-DFc and mFo-DFc omit maps for DNA region.**

1. **DNA double helix with chains C and D**

**
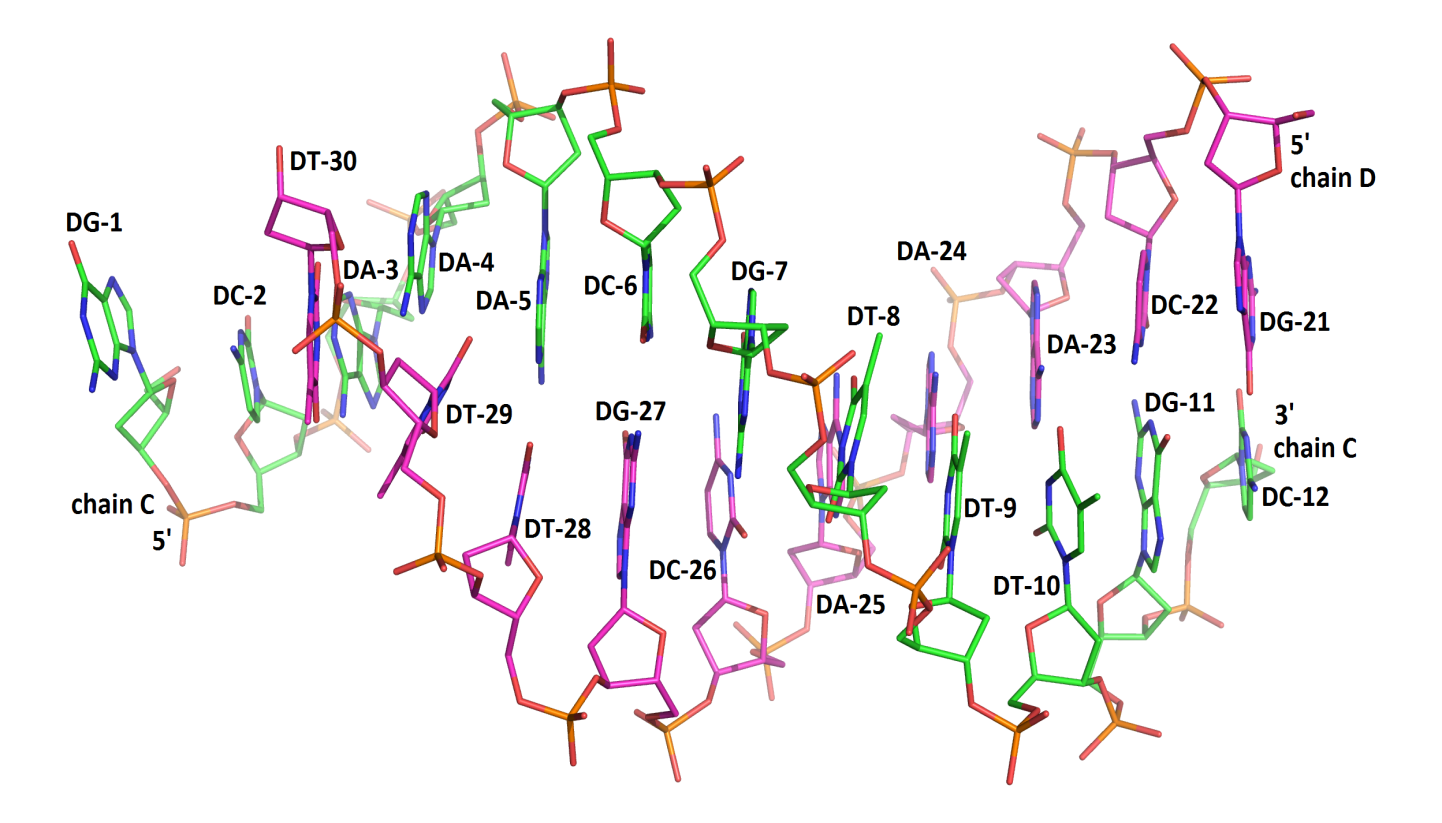
**

1. **2mFo-DFc map (contoured at 1.5σ level)**


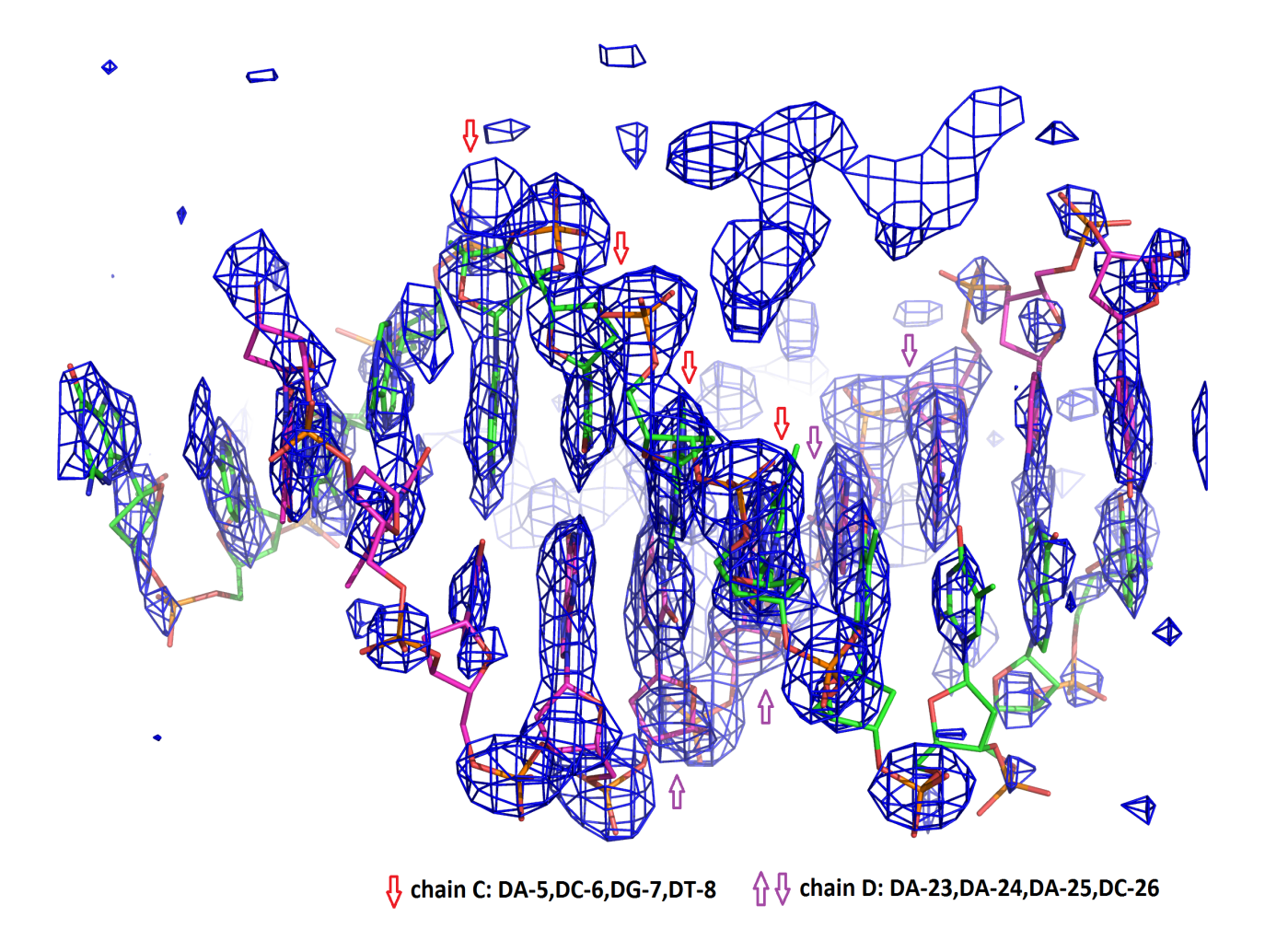


1. **mFo-DFc omit map (contoured at 2.5σ level)**


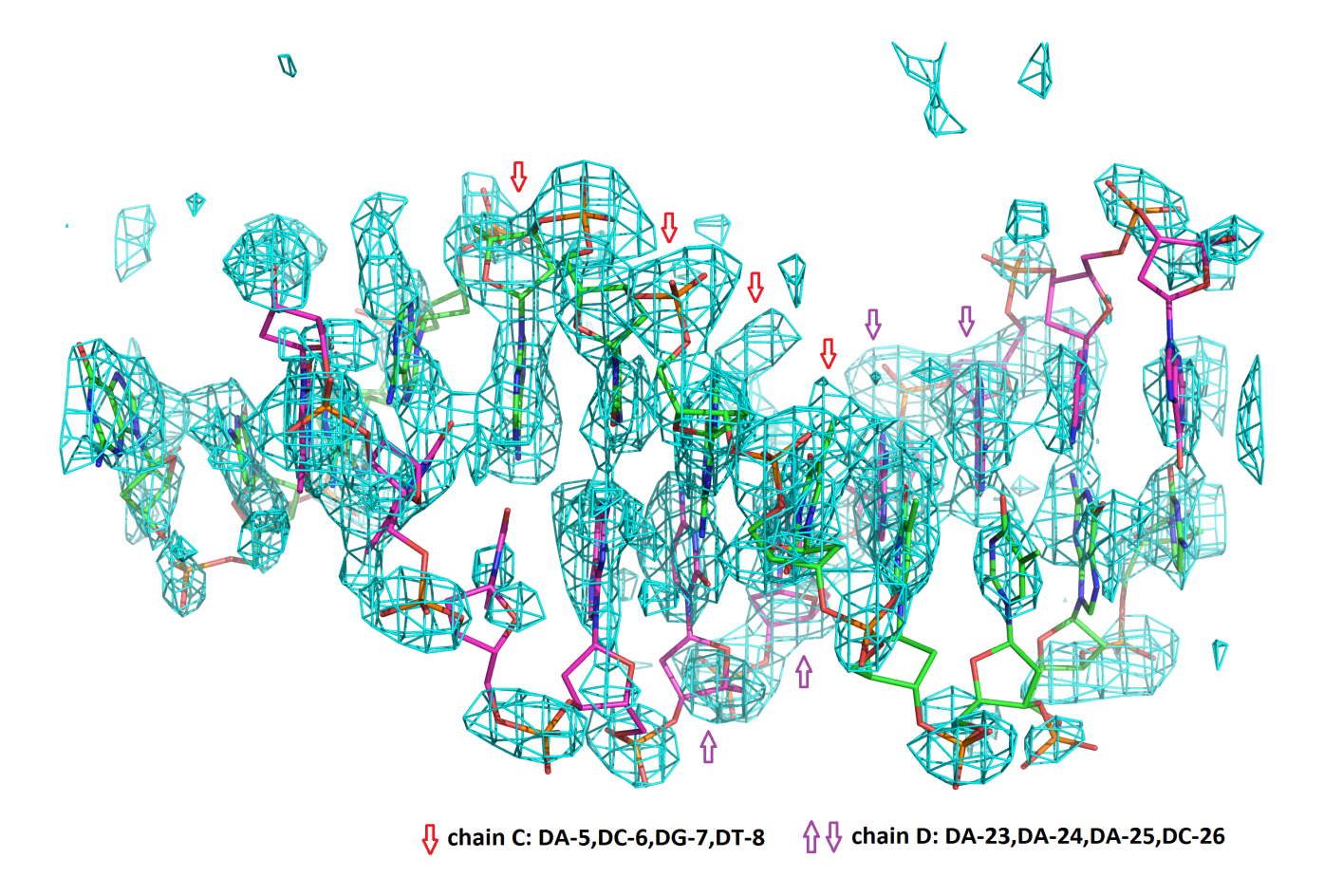


1. **2mFo-DFc simulated annealing omit map (contoured at 1.0σ level)**

**
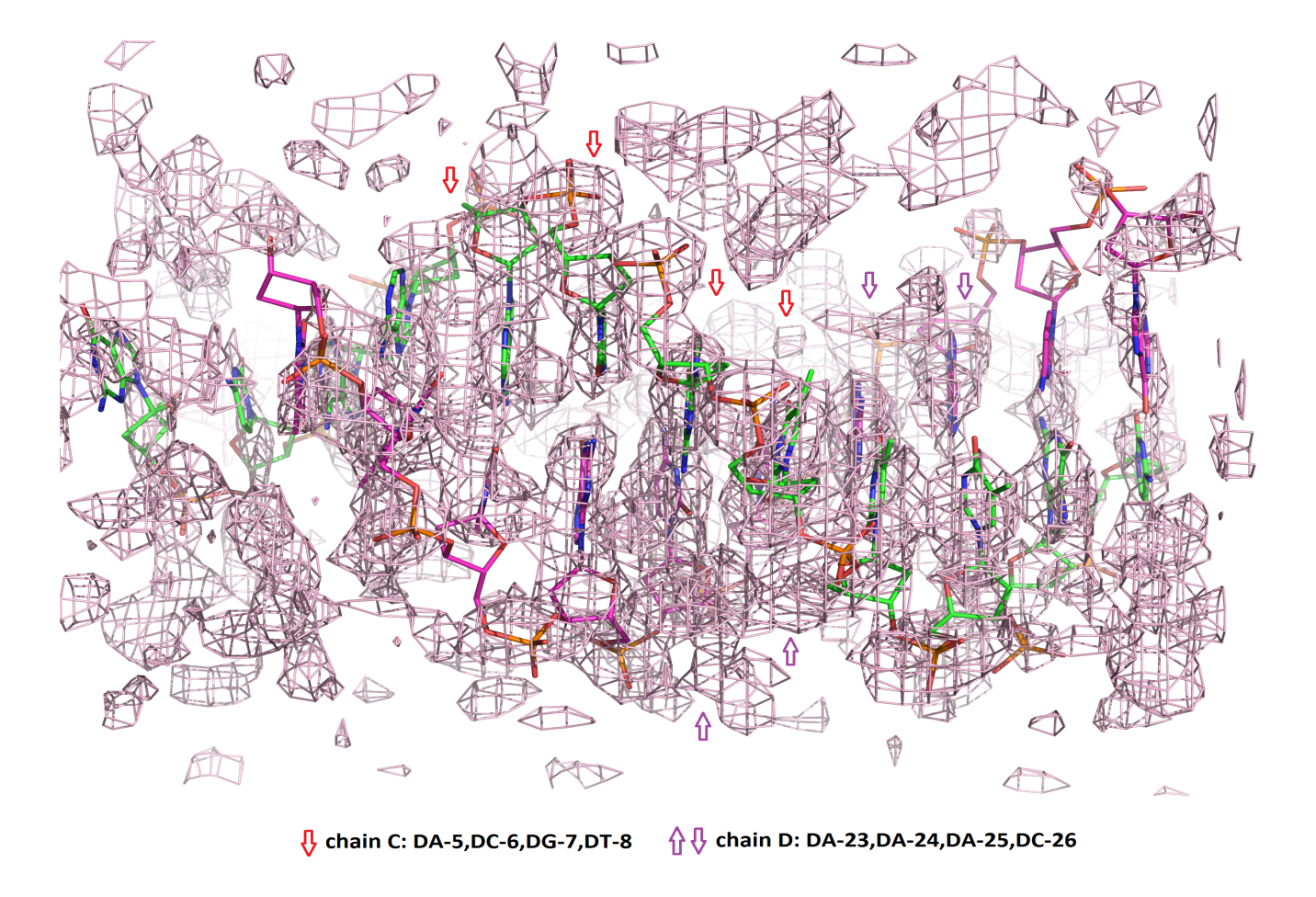
**

The 2mFo-DFc map (1.5σ contour level) is the sigma weighted map after final maximum likelihood refinement in REFMAC. The mFo-DFc omit map (2.5σ contour level) is an unbiased map generated by maximum likelihood refinement in REFMAC after removal of the DNA coordinates from the coordinate file. The 2mFo-DFc simulated annealing (SA) omit map (1.0σ contour level) is an unbiased composite map generated in PHENIX. This SA omit map is composed of 24 omit regions that include all 22 nucleotides of the DNA helix. DNA nucleotides are represented as stick models (chain C: C green, O red, N blue, P orange; chain D: C magenta, O red, N blue, P orange). The view in A-C of Figure S1 is the same. Termini (5’ and 3’) of DNA chains C and D and nucleotides in Figure S1A are labeled. The arrows highlight nucleotides 5-8 in chain C and 23-26 in chain D.

**Figure S2.** **SigmaA weighted 2mFo-DFc map for D4-DNA interfaces.**

1. **D4-DNA interface 1 (chains A and D).**


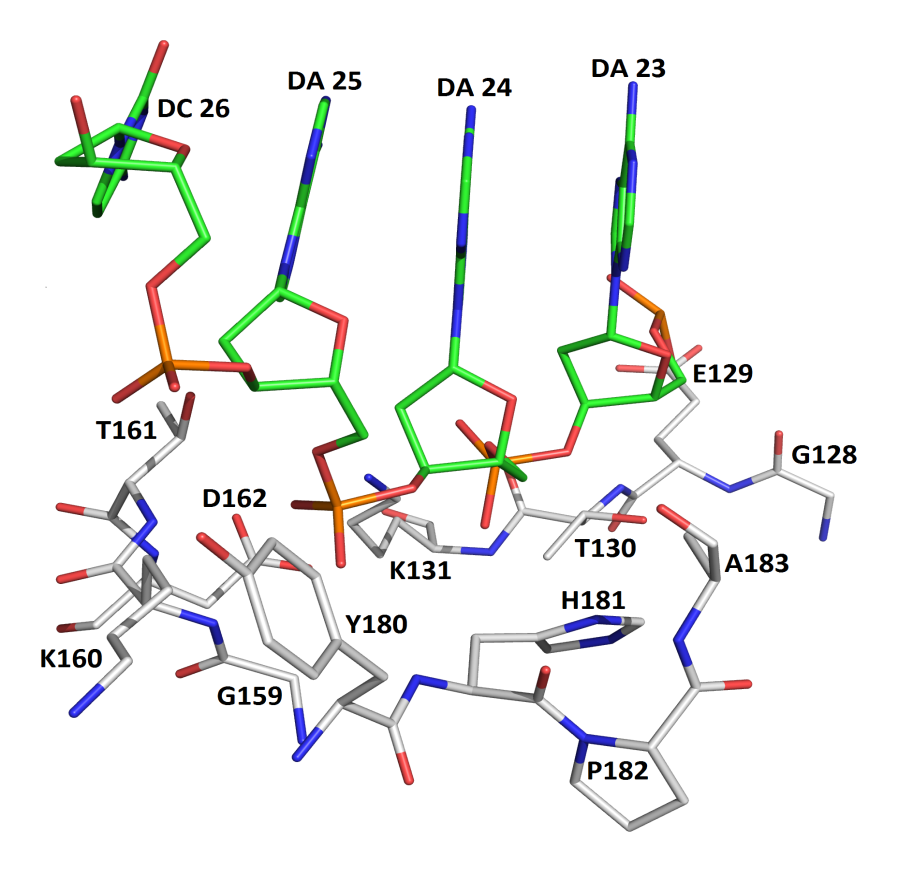


1. **D4-DNA interface 1 (chains A and D).**


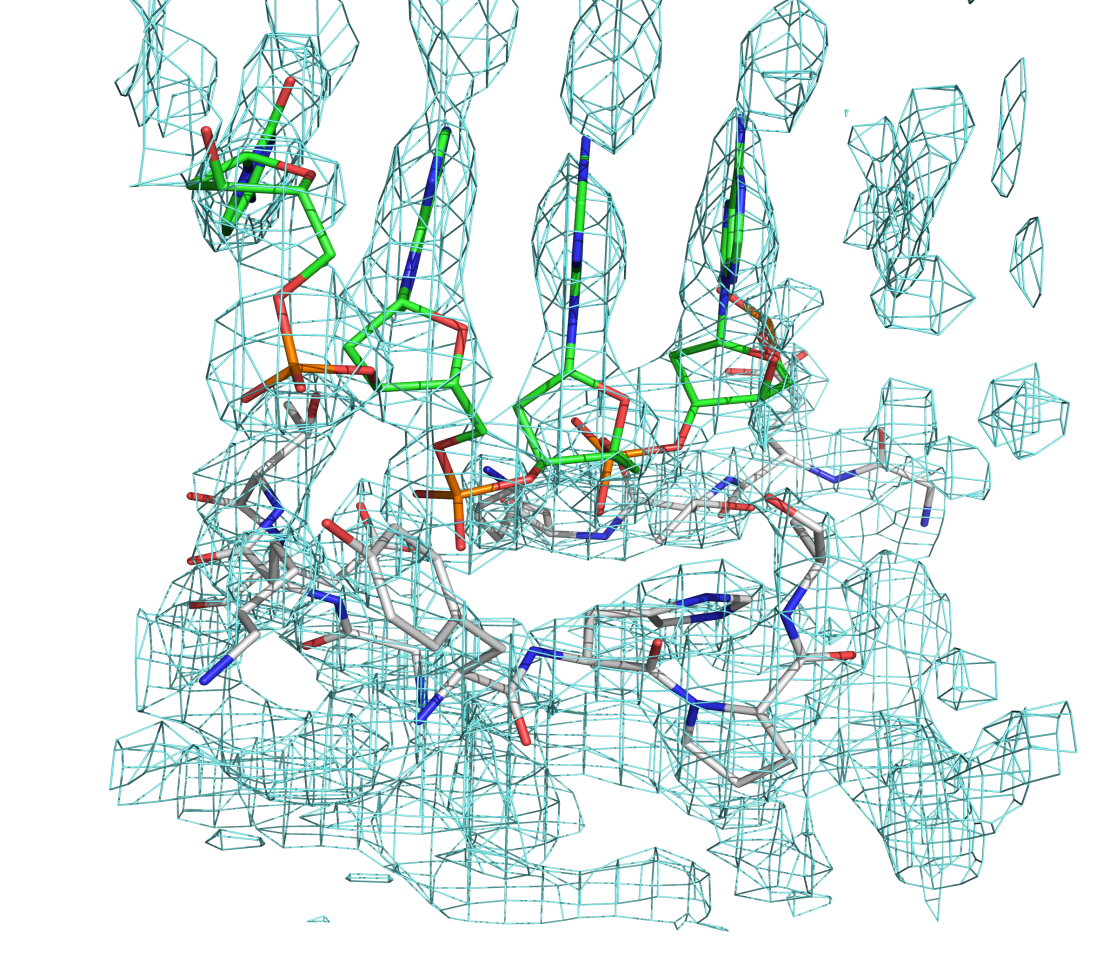


**C. D4-DNA interface 2 (chains B and C).**

**
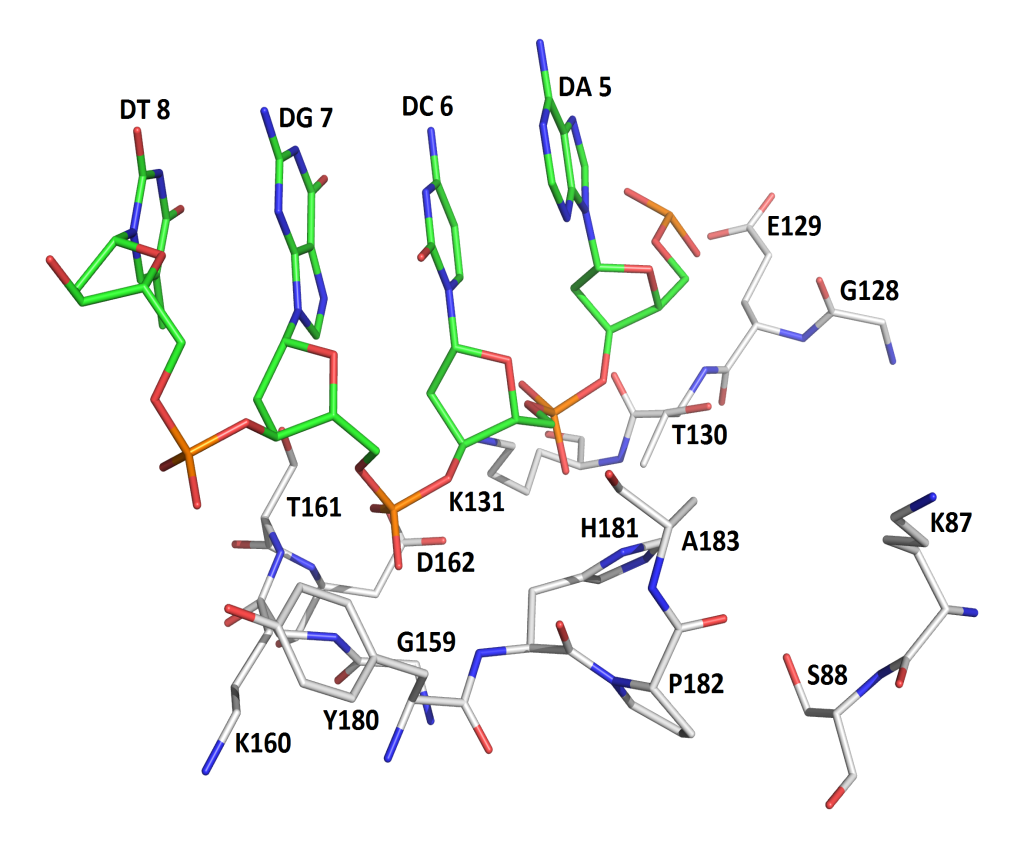
**

**D. D4-DNA interface 2 (chains B and C).**

**
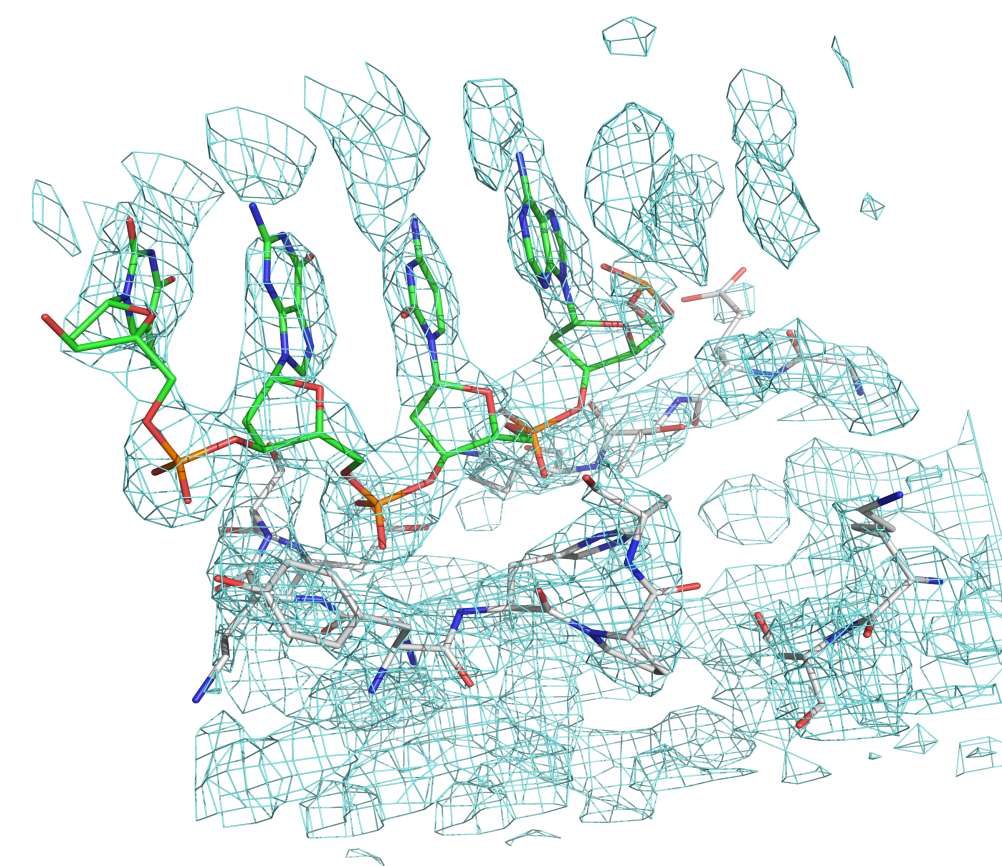
**

The 2mFo-DFc map (1.5σ contour level) in aquamarine color (**Fig. S2B and Fig. S2D**) for the D4-DNA interfaces is the sigma weighted map after final maximum likelihood refinement in REFMAC.

Views in **Fig S2A** & **S2B** as well as **S2C** & **S2D** are the same. Interacting DNA nucleotides (C green, O red, N blue, P orange) and D4 interface residues (C grey, O red, N blue) are shown as stick models. **Figs. 2A** & **2C** show the labeling of nucleotides and D4 residues.

**Figure S3. Packing of D4-DNA complex.**

**
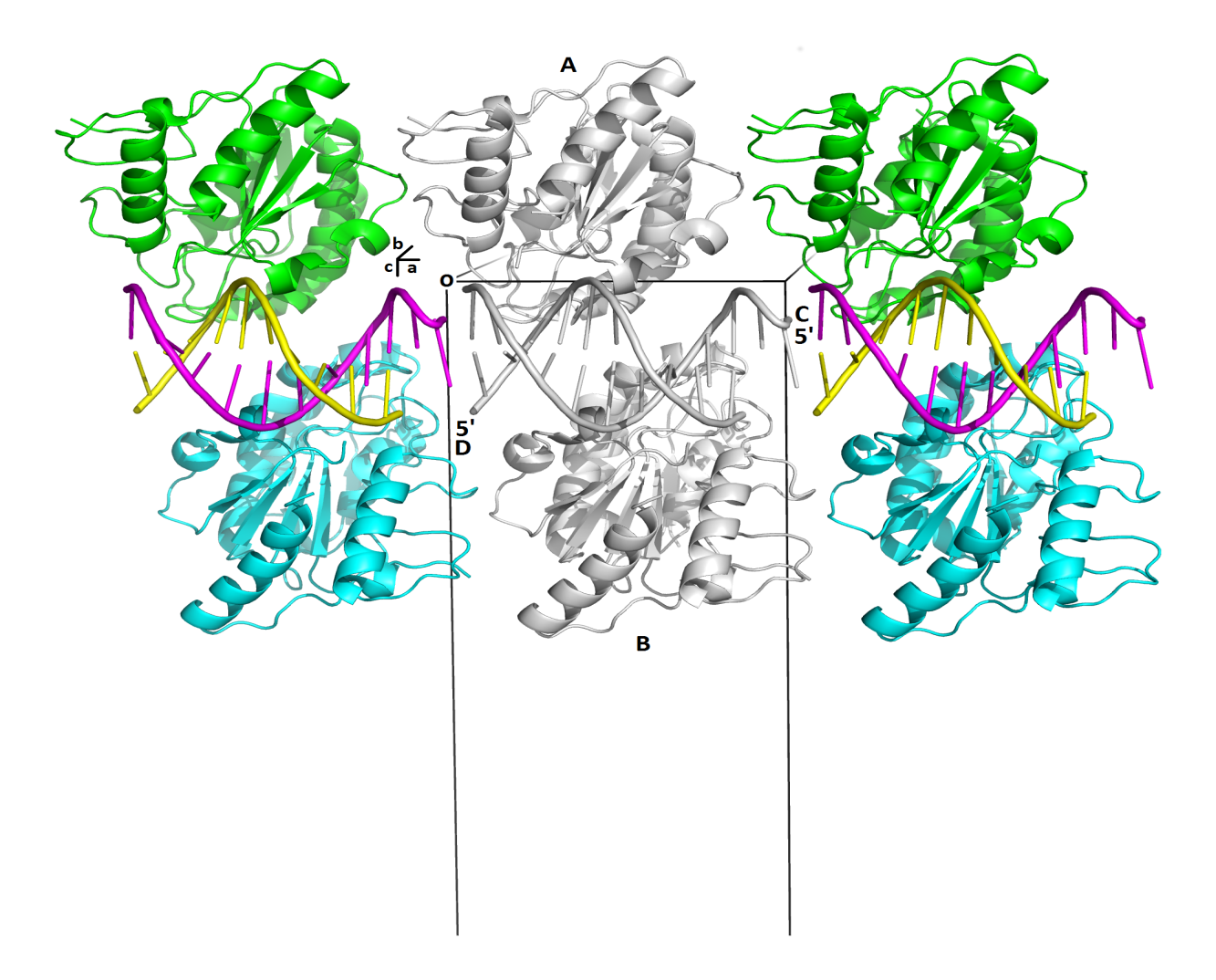
**

The figure highlights in the center the two D4 subunits in complex with the non-specific dsDNA construct. Left and right are symmetry-mates (along a) to emphasize how the blunt-end DNA helix extends in the unit cell through non-covalent interactions at the 5’ and 3’ ends. Protein and DNA are represented as cartoon drawings (symmetry-mates in chain color). Protein subunits A and B and DNA chains C and D are shown in grey. The 5’ end of each DNA strand is labeled.

The unit cell is outlined in black (origin O and direction of the unit cell parameters a, b and c are indicated)

All three figures were generated in PyMOL [7].

**Figure S4. Electron density map.**

**
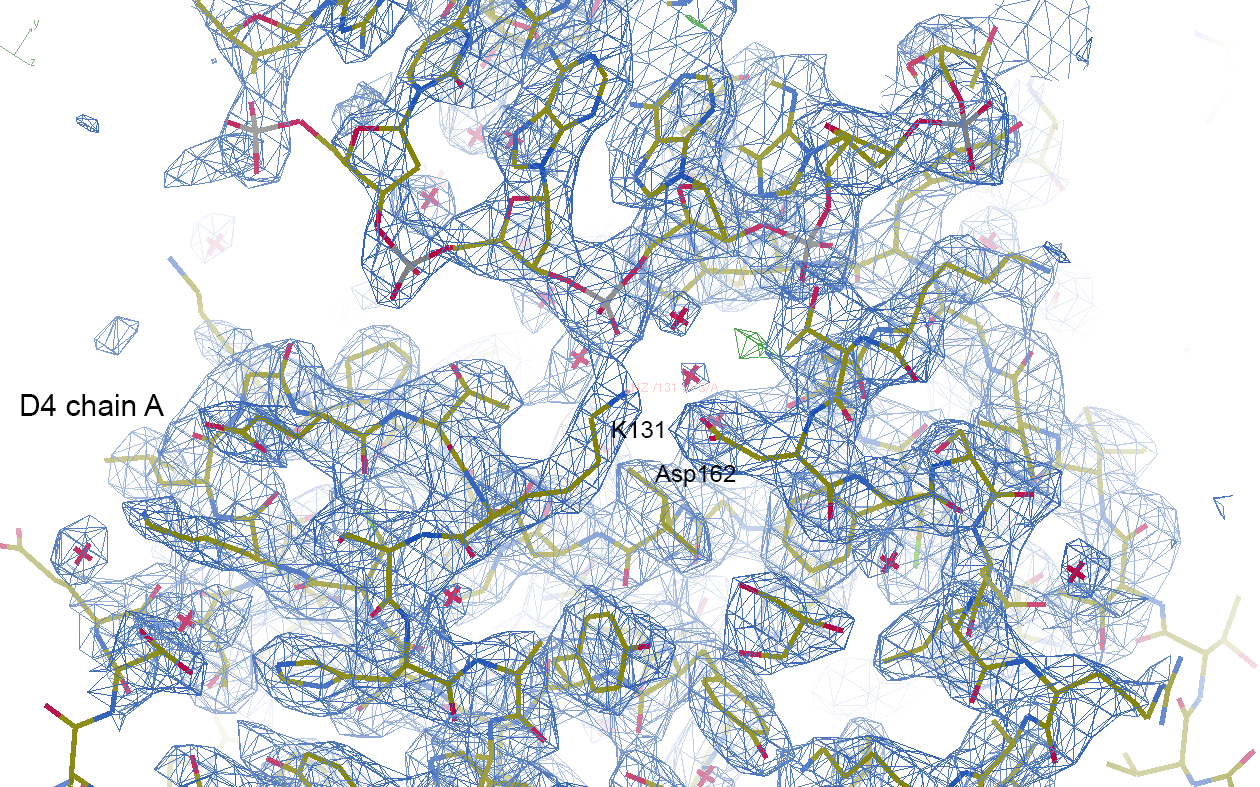
**

**
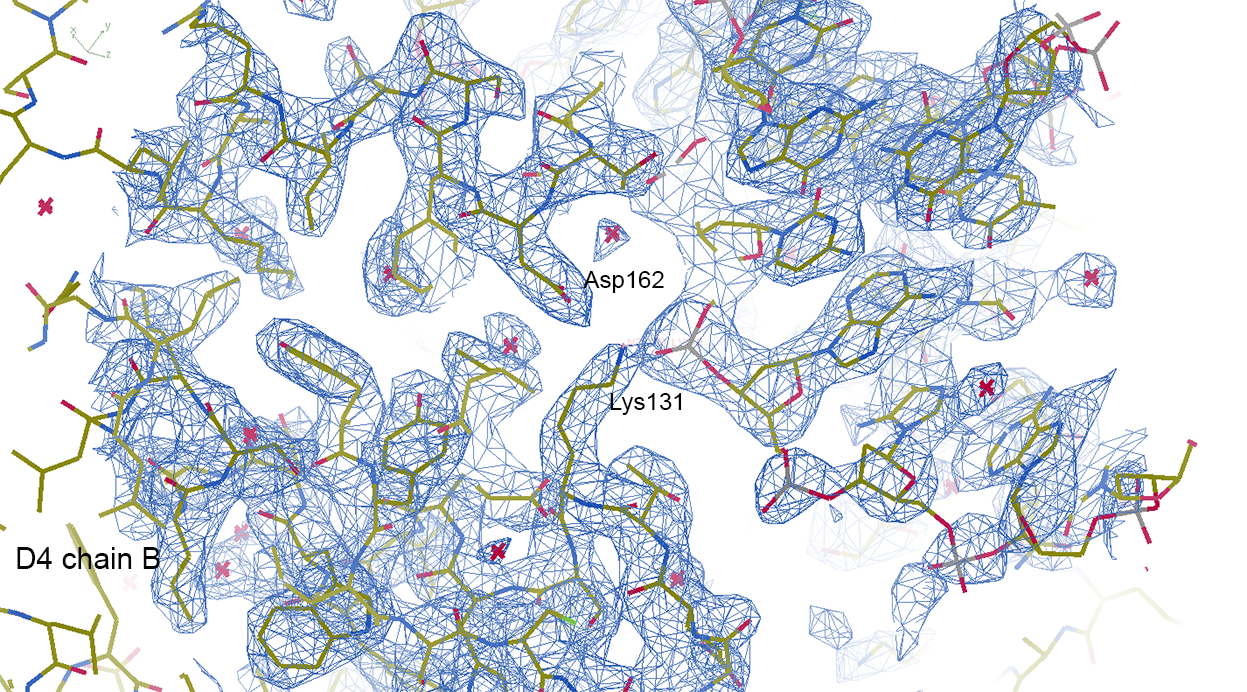
**

Electron density maps represent screenshots from Coot [8] showing final model and 2Fo-Fc electron density map contoured at 1.0σ. Amino acid residues are shown in stick model; Lys131 and Asp162 are labeled.

**References**

1. Viadiu H, Aggarwal AK. Structure of BamHI bound to nonspecific DNA: a model for DNA sliding. Mol Cell. 2000;5:889-895.
2. Parikh SS, Walcher G, Jones CD, Slupphaug G, Krokan HE, Blackburn GM, Tainer JA. Uracil-DNA glycosylase-DNA substrate and product structures: conformational strain promotes catalytic efficiency by coupled stereoelectronic effects. Proc. Natl. Acad. Sci USA. 2000;97:5083-5088.
3. Bianchet MA, Seiple LA, Jiang YL, Ichikawa Y, Amzel LM, Stivers JT. Electrostatic guidance of glycosyl cation migration along the reaction coordinate of uracil DNA glycosylase. Biochemistry. 2003;42:12455-12460.
4. Parikh SS, Mol CD, Slupphaug G, Bharati S, Krokan HE, Tainer JA. Base excision repair initiation revealed by crystal structures and binding kinetics of human uracil-DNA glycosylase with DNA. EMBO J. 1998;17:5214-5226.
5. Parker JP, Bianchet MA, Krosky DJ, Friedman, JI, Amzel LM, Stivers JT. Enzymatic capture of an extrahelical thymine in the search for uracil in DNA. Nature. 2007;449:433-437.
6. Slupphaug G, Mol CD, Kavli B, Arvai AS, Krokan HE, Tainer JA. A nucleotide-flipping mechanism from the structure of human uracil-DNA glycosylase bound to DNA. Nature. 1996;384:87-92.
7. DeLano WL. PyMOL. 2002. (Schrödinger, LLC; Open-Source PyMOL^TM^, version 1.7.x).
8. Emsley P, Cowtan K. Coot: Model-building tools for molecular graphics. Acta Cryst D Biol Cryst. 2004;60:2126-2132.
